# Recent Advances in Monitoring Technologies for Cardiac Troponin I: A Pivotal Biomarker in Cardiovascular Diseases

**DOI:** 10.3390/biom15060858

**Published:** 2025-06-12

**Authors:** Ning Zhang, Yusong Wang, Fachuang Li, Yuxin Zhu, Zheng Fu, Mengda Jia, Xiaoran Zhan, Wanqing Zhang

**Affiliations:** 1 School of Materials Science and Engineering, Henan Institute of Technology, Xinxiang 453003, China; znzhangning121@hait.edu.cn (N.Z.); cwd818@hait.edu.cn (F.L.); 2 School of Chemistry and Chemical Engineering, Henan Institute of Science and Technology, Xinxiang 453003, China; wangys@stu.hist.edu.cn (Y.W.); zhuyx@stu.hist.edu.cn (Y.Z.); jmd0519@stu.hist.edu.cn (M.J.); zhanxiaoran@stu.hist.edu.cn (X.Z.)

**Keywords:** cTnI, sensor, optical technology, electrical technology, intelligent technology

## Abstract

Cardiovascular diseases (CVDs) are among the leading causes of morbidity and mortality rates globally, presenting a severe threat to human health and life. Acute myocardial infarction (AMI) is one of the most common and extremely severe disorders within CVDs, causing an estimated 17.5 million deaths each year. Cardiac troponin I (cTnI) is considered a biomarker for myocardial infarction and a “gold standard” method for diagnosing AMI due to its high specificity and sensitivity. The ability to rapidly detect cTnI with high sensitivity is critical throughout the diagnosis and treatment process of AMI. It is a necessary precursor for doctors to quickly assess the disease and initiate subsequent therapies. This work comprehensively explores various techniques for the analysis and detection of cTnI. We systematically review current cutting-edge technologies used for cTnI detection. According to optical, electrical, and intelligent technology dimensions, this study meticulously classifies and elaborates on the research progress of related sensors. Based on current research findings and technological development trends, we further project the future research directions and application prospects of cTnI sensors. This is geared towards providing valuable references for the further development of this field.

## 1. Introduction

Cardiovascular diseases (CVDs) are a group of disorders that severely affect the human heart and blood vessels [1,2,3,4,5,6,7,8]. They are the most common disease category across the globe and the leading cause of human death [6,9,10,11,12,13,14]. Various unhealthy lifestyles and habits can significantly increase the risk of CVDs [15,16,17,18,19]. Among them, unhealthy diet, smoking, obesity, and lack of exercise are the major risk factors for CVDs. In 2024, the World Heart Federation reported that an estimated 18 million people globally succumbed to CVDs, accounting for 31% of the global annual total deaths. This shows the severe threat of CVDs to human health [20,21,22,23,24,25,26,27].

Acute myocardial infarction (AMI) is an extremely prevalent type within CVDs [28,29,30,31,32,33,34]. It occurs when the myocardium becomes damaged once the coronary artery is blocked, causing insufficient blood supply (i.e., ischemic state). Myocardial necrosis is irreversible [35,36,37,38]. Studies indicate that up to 85% of heart damage occurs within the first two hours following a heart attack. Therefore, developing early and accurate detection methods for AMI is critical to establishing accurate diagnosis, prompt treatment, and effectively improving survival rates [39,40,41,42,43,44]. Determining whether AMI has occurred depends on measuring the concentration of biomarkers in blood samples [45,46,47,48,49,50,51]. Cardiac troponin I (cTnI), cardiac troponin T (cTnT), C-reactive protein (CRP), etc., are the most important biomarkers for AMI. Unlike other biochemical markers of myocardial injury including creatine kinase-MB and myoglobin, cTnI has extremely high cardiac specificity and is a specific marker for coronary artery events [52,53,54,55,56,57]. Therefore, accurate detection of cTnI has been a research focus in multiple fields over recent years, hence bringing breakthroughs to the clinical diagnosis of CVDs [58,59,60,61,62,63,64,65].

Several techniques for detecting cTnI have been developed, including optical [66], electrical [67], and other intelligent detection methods. In the field of optical detection, immunofluorescence analysis (IFAL) [68] uses fluorescently labeled antibodies specifically binding to cTnI. Under excitation light irradiation fluorescent labels emit fluorescence. The concentration of cTnI can be determined by accurately detecting fluorescence intensity. This technique can yield highly sensitive detection of cTnI since the fluorescence signal is easy to capture [69,70,71].

Enzyme-linked immunosorbent assay (ELISA) [52,72], which is a cornerstone of immunodetection, relies on the specific antigen–antibody interaction. Antigens or antibodies are immobilized on solid-phase carriers like polystyrene microplates before adding a sample containing cTnI and an enzyme-labeled antibody. Thereafter, an antigen–antibody–enzyme complex is formed after incubation and washing to remove unbound components. The addition of a substrate triggers enzyme-catalyzed color development; absorbance changes are then measured to accurately quantify cTnI. ELISA is extensively integrated into clinical diagnostics due to its technical maturity and operational simplicity.

Surface-enhanced Raman scattering (SERS) [73] detection relies on the exponential amplification of Raman signals of molecules adsorbed onto engineered metal nanostructures (e.g., gold or silver nanoparticles). For cTnI detection, SERS-active nanoparticles are conjugated with cTnI-specific antibodies. Minute Raman signal alterations are sensitively detected upon specific binding to cTnI in the sample, enabling ultra-trace quantification of cardiac troponin. This exceptional sensitivity is critical for early diagnosis of CVDs, where timely detection of low-abundance biomarkers is important.

Surface plasmon resonance (SPR) [74] technology uses a principle of surface plasmon wave generation when incident light strikes a metal–medium interface at a resonant angle, exciting free electrons in the metal. In cTnI analysis, anti-cardiac troponin I antibody (anti-cTnI) antibodies are immobilized on the metal surface. The binding of cTnI from the sample causes the refractive index to change at the interface, shifting the SPR angle or wavelength. Accurate measurement of these optical changes allows label-free, real-time quantification of cTnI with high specificity.

Fiber-optic sensor technology (FOST) [75] adopts the electromagnetic interference resistance, high sensitivity, and long-range transmission properties of optical fibers to detect cTnI in complex matrices. This platform ensures stable and adaptive performance in real-world applications, thus providing reliable diagnostic data. Conversely, photoelectrochemical sensing monitors photocurrent or photovoltage fluctuations generated by light-excited redox reactions. Through optimized sensor design, it captures subtle electrical signal variations correlating with cTnI concentration, hence providing a novel and effective detection paradigm.

In electrochemical detection, electrochemical immunosensing (EIT) [76] combines the sensitivity of electrochemical analysis with the specificity of antigen–antibody binding. Immobilizing recognition elements on electrode surfaces forms a platform where target binding causes measurable changes in electrical signals (e.g., current or impedance). EIT is transformative for early AMI diagnosis as it is capable of detecting cTnI at picogram-per-milliliter levels. Electrochemical aptamer detection (EADT) utilizes synthetic single-stranded DNA/RNA aptamers with a high-affinity for cTnI recognition. Upon target binding, immobilized aptamers on electrodes undergo conformational changes, hence changing electrochemical properties that are subsequently transduced into quantifiable signals. The inherent specificity of aptamers reduces interference, thereby improving diagnostic reliability.

Field-effect transistors (FETs) [77] and voltage-controlled semiconductors provide unique benefits in label-free cTnI sensing. Antibodies functionalized on the FET gate surface bind with cTnI, causing charge redistribution that regulates electrical parameters like source-drain current or threshold voltage. Sensitive detection of these changes enables rapid quantitative analysis with high sensitivity, hence supporting point-of-care (POC) applications.

Electrochemiluminescence immunoassay (ECLIA) [78] integrates electrochemiluminescence with immunoassay, where luminophore-labeled antibodies emit photons upon redox cycling at electrode surfaces. Signal intensity, proportional to cTnI concentration, is detected with high accuracy, enabling batch processing and rapid turnaround suitable for clinical laboratories handling diverse patient needs.

Emerging technologies including lateral flow immunoassay [79], microfluidics [80], and intelligent sensing [81] further improve cTnI detection. Lateral flow assays allow rapid semi-quantitative POC screening via intuitive test strips, whereas microfluidics miniaturizes workflows onto microchips, hence reducing reagent consumption and analysis duration for high-throughput testing. Intelligent sensing, which combines nanomaterials and biosensors, promises improved sensitivity and specificity, providing a reference for next-generation diagnostic tools.

This review systematically looks into optical, electrical, and hybrid sensor platforms for cTnI detection, emphasizing principles, methodologies, and recent point-of-care testing (POCT) advancements. Technological breakthroughs address persisting challenges, including high costs limiting scalability, portability constraints for on-the-go use, and reliability issues in complex biological environments. Compared with traditional review literature, which tends to focus on descriptions of experimental data and interpretations of partial mechanisms, as well as introducing technical pathways for constructing cTnI recognition sensors using different materials, there is a general lack of attention to the impact of actual sample matrix differences (especially the distinction between clinical samples and calibrated standard solutions) on detection performance. Based on a systematic review of relevant content, this review not only focuses on the above-mentioned matrix type issues but also elaborates in detail on key problems such as the selectivity and cross-reactivity mechanisms of sensors, the regulatory role of biological receptors (such as antibodies and aptamers) on sensor performance, and the reliability assessment of detection limits and detection ranges based on clinical samples. Looking forward, interdisciplinary innovations in nanotechnology and biomedical engineering are anticipated to drive the development of more precise, rapid, and accessible cTnI sensors, hence revolutionizing early disease detection and improving global healthcare.

## 2. Optical Detection Methods

Immunofluorescence analysis (IFAL), enzyme-linked immunosorbent assay (ELISA), surface-enhanced Raman scattering (SERS), surface plasmon resonance (SPR), fiber-optic sensing technology (FOST), and photoelectrochemical (PEC) sensing are the current major optical techniques for detecting cTnI.

Notably, IFAL has the peculiar benefit of intuitive visualizability. Using a fluorescence microscope, researchers can directly and accurately observe the binding state between fluorescently labeled antibodies and cTnI, a feature that significantly simplifies the detection process and enables its widespread use in cTnI testing. ELISA, which is supported by a mature technical system, guarantees high detection accuracy by constructing rigorous standard curves and strict experimental operation control, hence making it a common and reliable immunoassay for clinical cTnI detection.

SERS is minimally affected by fluorescent background interference and provides rich molecular structural information, allowing accurate identification and detection of cTnI in complex biological samples at the same time, effectively preventing interference from other substances. The SPR technique allows label-free direct detection and real-time tracking of biomolecular interactions, which is critical for early diagnosis of AMI, helping disease detection at its onset and promptly intervening to secure treatment.

FOST optimizes optical fiber’s electromagnetic interference resistance, high sensitivity, and long-distance transmission properties for precise cTnI detection in complex environments, thereby demonstrating excellent stability and adaptability in practical applications. PEC technology uses photocurrent or photovoltage changes induced by light excitation. Through a carefully designed sensing system, PEC sensitively captures photoelectric signals induced by variations in cTnI concentration, hence opening a novel and efficient pathway for cTnI detection. Each of these optical methods has unique benefits, collectively driving continuous innovation and development in cTnI detection technologies.

### 2.1. Immunofluorescence Assay (IFAL)

**Figure 1:**
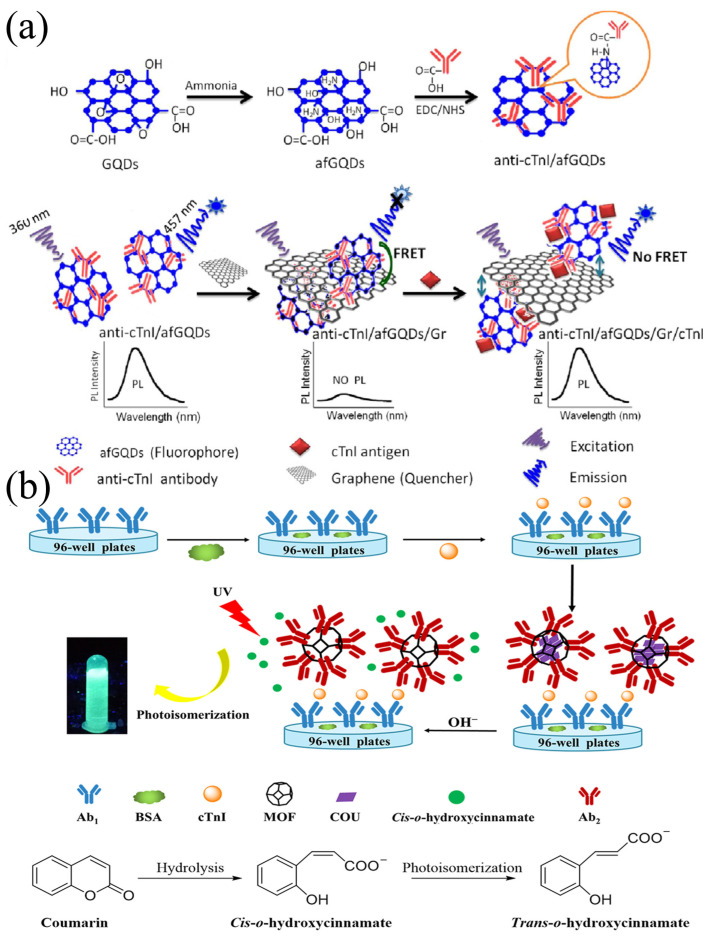
(**a**) Construction of anti-cTnI/afGQDs; (**b**) MOF@COU/Ab2 immunosensors and the mechanisms of their recognition of cTnI. (**a**) reproduced from Ref. [82], Copyright 2018, ScienceDirect. (**b**) reproduced from Ref. [83], Copyright 2023, ScienceDirect.

Ashok Kumar et al. [82] prepared amine-functionalized graphene quantum dots (afGQDs) combined with anti-cTnI. The prepared anti-cTnI/afGQDs immunosensor could detect the antigen cTnI in serum within 10 min because of the fluorescence resonance energy transfer (FRET) between the conjugate and graphene (quencher) (Figure 1a). The sensor possessed excellent performance: the linear detection range for cTnI was 0.001–1000 ng/mL and the limit of detection (LOD) was as low as 0.192 pg/mL. Additionally, the sensor exhibited high specificity, with negligible cross-reactivity to common coexisting substances such as myoglobin and CRP.

Using a hydrothermal synthesis method, Xiaoying Wang et al. [83] prepared zeolitic imidazolate framework-8 (ZIF-8) material with high porosity and a large specific surface area. A secondary antibody (Ab2) specific to cTnI was coated on the surface of this material after integrating it with an antibody and a fluorescent probe coumarin (COU). The prepared MOF@COU/Ab2 composite material achieved an enzyme-free detection of cTnI (Figure 1b). Serum samples were directly detected by fluorescence immunoassay without dilution. After antibody-coated 96-well plates were incubated and washed, they were blocked with bovine serum albumin (BSA), loaded with samples for incubation, and then MOF@COU/Ab2 was added for incubation. After washing, NaOH solution was added to measure the fluorescence intensity and each sample was measured three times. The immunosensor had a wide linear range (11.1 fM–35.6 pM) and a low LOD (0.099 pg/mL). At a cTnI concentration of 0.1 ng/mL, the fluorescence intensity showed a difference of less than 5% when compared to that in a mixture containing 10 ng/mL Carcinoembryonic Antigen (CEA), Alpha Fetoprotein (AFP), and C-reactive protein (CRP), indicating good selectivity of the sensor.

Kohji Mitsubayashi et al. [84] developed a surface plasmon-enhanced fluorescence sensor device for the dynamic detection of cTnI. This device uses a semi-continuous measurement mode with a surface modification technology that can regenerate the sensor. This sensor system is based on the principle of attenuated total reflection at a wavelength of 633 nm in the Kretschmann structure. The gold substrate is specially modified and a stable scaffold protein fused with protein G is attached to it to achieve immobilization and reuse of the capture antibody. cTnI is detected using a sandwich method, with a detection range of 3.9–100 ng/mL and an LOD of 0.98 ng/mL. The detection speed of this method is six times faster than that of ELISA.

Elsewhere, Sony George et al. [85] used Mn^2+^-doped ZnS quantum dots coated with mercaptopropionic acid (MPA) and MnO_2_ nanosheets as a fluorescence resonance energy transfer (FRET) pair to construct a MnO_2_-Ab@MQD fluorescent immunosensor for detecting cTnI. The LOD of this sensor in identifying cTnI was 0.05 ng/mL. This sensor obtained a good recovery rate in the detection of actual serum samples, thereby demonstrating its application for the point-of-care testing of cTnI.

### 2.2. Enzyme-Linked Immunosorbent Assay (ELISA)

**Figure 2:**
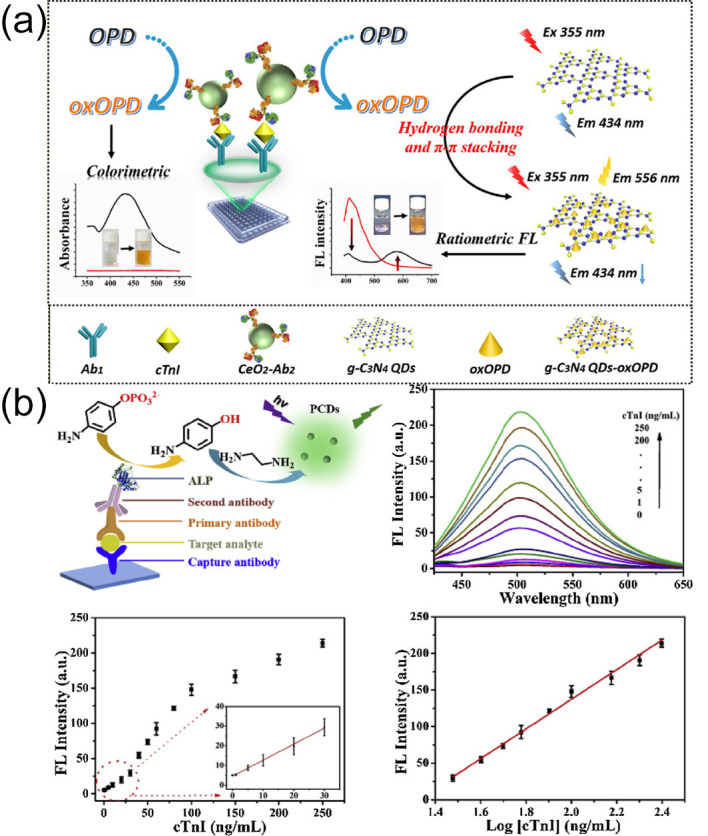
(**a**) Schematic illustration of constructing a multimodal immunoassay platform based on g-C_3_N_4_ quantum dots–CeO_2_ nanozyme conjugation and its recognition mechanism for cTnI; (**b**) the detection mechanism of ALP-triggered fluorescence ELISA for cTnI. (**a**) reproduced from Ref. [86], Copyright 2019, ScienceDirect. (**b**) reproduced from Ref. [87], Copyright 2020, ScienceDirect.

He Li et al. [86] prepared graphitic carbon nitride quantum dots (g-C_3_N_4_ QDs) and employed them as an exogenous fluorescent signal source. In a system with o-phenylenediamine (OPD) and H_2_O_2_, making use of the peroxidase-like capacities of CeO_2_ enzyme, OPD will be oxidized to 2,3-diaminophenazine (oxOPD), which has a maximum emission peak at 578 nm. Notably, oxOPD can effectively quench the corresponding fluorescence due to hydrogen bonding and π-π stacking interactions, causing a dual-emission peak fluorescence spectrum line under the irradiation of a single excitation wavelength. Additionally, the generation process of oxOPD is accompanied by a color change in the system for colorimetric detection. Based on the above principles, this team developed a nano-ELISA multimodal immunoassay platform. This platform can simultaneously perform dual-mode sensing of ratio fluorescence and colorimetry. In addition, it can precisely determine cTnI within a concentration range of 1 pg/mL–10 ng/mL. Figure 2a shows its detection mechanism.

Xiurong Yang et al. [87] used the Schiff base reaction, Michael addition reaction, and self-polymerization reaction between 4-aminophenol (AP) and ethylenediamine (EA). Green fluorescence and polymer carbon dots (PCDs) were produced during this reaction process. Additionally, alkaline phosphatase (ALP) can catalytically convert 4-aminophenyl phosphate (APP) into AP specifically, and EA can differentiate between AP and APP. Combined with conventional ELISA, a novel ALP-triggered fluorescence ELISA was established through the in situ production of PCDs. As a result, we successfully achieved quantitative detection of cTnI in human serum (with a concentration range of 1–250 ng/mL) (Figure 2b). The study used clinical real serum samples (including those from healthy individuals and cardiovascular disease patients) for verification. The results showed that the cross-reactivity to coexisting biomarkers (such as myoglobin and CRP) was less than 5%, confirming excellent selectivity. Furthermore, the sensor retained more than 92% of its fluorescence signal after storage at 4 °C for 30 days, and the relative standard deviations (RSDs) for intra-batch and inter-batch detections were less than 4.8%, demonstrating good stability and reproducibility.

Jian Sun et al. [88] carried out a specific reaction between ascorbic acid (AA) and N-methyl ethylenediamine (N-MEDA) under mild conditions to produce fluorescent non-conjugated polymer dots (NCPDs). This research group developed a fluorescence technique for detecting ALP activity based on AA-responsive fluorescence emission and the fact that alkaline phosphatase (ALP) catalyzes the hydrolysis of ascorbic acid 2-phosphate to produce AA. In combination with the classical ELISA method we selected cTnI as the model target, with a linear detection range of 0–100 ng/mL.

Using a wavelength-tunable fluorescence immunoassay method caused by copper ions (Cu^2+^), Jinhua Liu et al. [89] developed an “on-off-on” strategy platform for real-time detection of cTnI in human serum. This method primarily involves the in situ fluorescence generation reaction of dopamine (DA) and phenolic analogs under Cu^2+^, the strong coupling effect of pyrophosphate (PPi) and Cu^2+^, as well as the ALP process specifically hydrolyzing PPi into orthophosphate. Subsequently, this research group used cTnI as a model antigen and different phenolic analogs (1,3-dihydroxynaphthalene, 8-hydroxyjulolidine, and 1,5-dihydroxynaphthalene) as fluorescent reaction substrates. This was to achieve multi-channel (blue, green, and yellow) real-time monitoring of cTnI, with LODs of 0.17 ng/mL, 0.17 ng/mL, and 0.33 ng/mL, respectively.

### 2.3. Surface-Enhanced Raman Scattering (SERS)

Lingxin Chen et al. [90] utilized gold nanoparticles (AuNPs), graphene oxide (GO), and magnetic beads (MBs). Through the interaction among antibodies and antigens they prepared a sandwich-type immunocomplex of “capture probe/target/SERS nanolabel” and developed a signal-amplified SERS platform for detecting cTnI. In this composite material the molecularly labeled AuNP-functionalized GO can be used as a SERS nanolabel and a signal amplification carrier. Notably, the monoclonal antibody-modified MB is used as a capture probe and a separating agent. The SERS immunoassay platform can perform molecular recognition of cTnI within the range of 0.01–1000 ng/mL and an LOD of 5 pg/mL.

**Figure 3:**
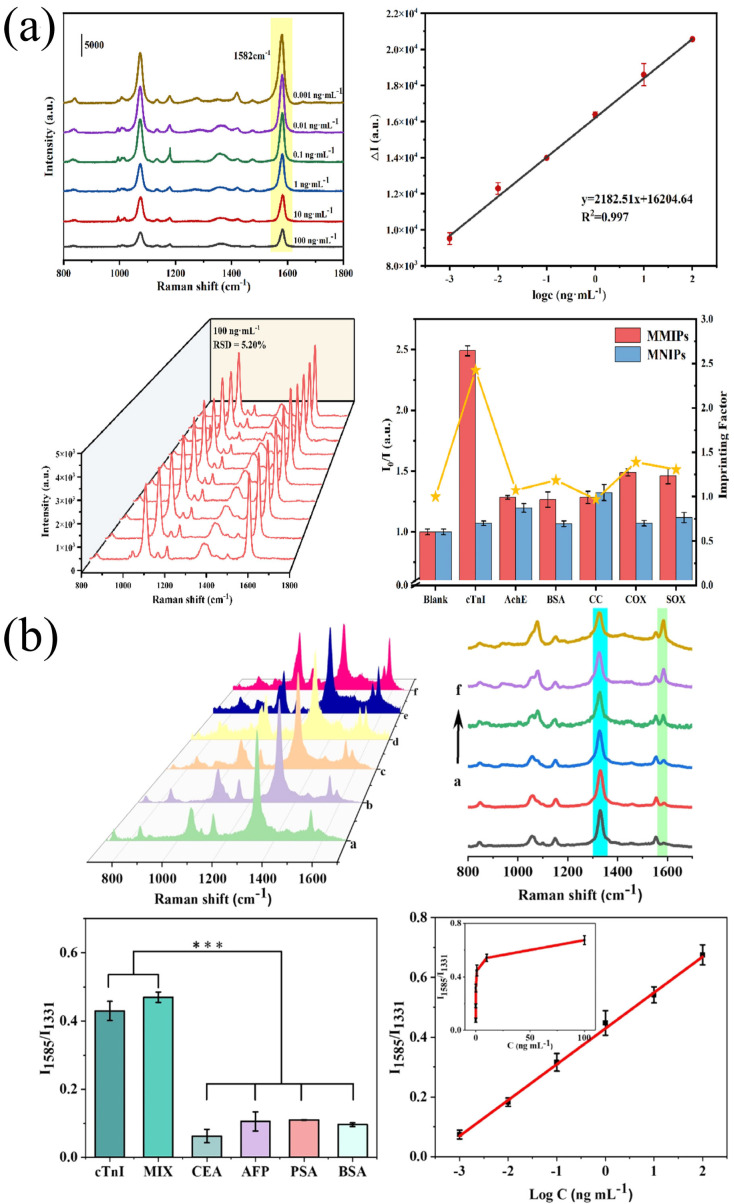
(**a**) Schematic illustration of standard curve construction and performance characterization for cTnI detection using Fe_3_O_4_-ZrO_2_-based SERS technology and (**b**) schematic diagram of the performance of alkaline phosphatase (ALP)-triggered fluorescence ELISA for the recognition of cTnI. (**a**) reproduced from Ref. [91], Copyright 2024, ScienceDirect. (**b**) reproduced from Ref. [92], Copyright 2024, ScienceDirect. *** *p* < 0.001.

Yong Liang et al. [91] coated a ZrO_2_ shell layer around the Fe_3_O_4_ magnetic core using the sol–gel method. Oxygen and hydroxyl groups were used to deposit silver nanoparticles on the surface of ZrO_2_. In addition, the Raman probe 4-mercaptobenzoic acid (4-MBA) was introduced through the Ag-SH bond to output the Raman signal. The shielding effect of the template peptide made the Raman signal inversely proportional to the template peptide concentration. Notably, the prepared SERS magnetic molecularly imprinted material can be utilized for the rapid quantitative detection of cTnI in human serum. The SERS had a detection range of 0.001–100 ng/mL and an LOD of 0.063 pg/mL (Figure 3a). The study used clinical real serum samples (including those from healthy individuals and myocardial infarction patients) to verify performance. Spike recovery rates ranged from 103.1% to 106.3%; cross-reactivity to structural analogs (such as cTnT and myoglobin) was less than 3.5%; after 10 cycles of reuse, the signal attenuation of the material was less than 6%; and the RSD for inter-batch detection was less than 4.5%. Together these confirmed its excellent anti-interference capability, stability, and reproducibility.

Shan-Shan Li et al. [92] adopted the gold array technique, in which they used 5,5′-dithiobis (2-nitrobenzoic acid) (DTNB) labeling as the SERS signal and modified the highly specific aptamer Tro4 onto it. The constructed SERS ratio-type aptamer sensor could specifically capture the cTnI substrate. The aptamer Tro6, targeting cTnI and 4-mercaptobenzoic acid (4-MBA), was anchored onto the porous gold (pAu) as another SERS signal to form the pAu signal probe. Upon addition of cTnI the captured substrate and the pAu signal probe can form a sandwich structure, making the SERS peak intensity ratio (I_4-mBa_/I_DTNB_) effective for quantitative detection of cTnI. The sensor exhibited excellent performance in human serum environments: its detection of cTnI spanned six orders of magnitude (0.001–100 ng/mL) and achieved a sensitivity of 0.27 pg/mL (Figure 3b). Validation using multi-center clinical serum samples (including AMI patients and healthy controls) showed that cross-reactivity to coexisting markers such as cTnT and myoglobin was less than 4.1%, that the inter-batch RSD for 15 consecutive serum detections was less than 4.9%, and that the signal variation in the probe solution remained within ±5.2% after storage at 4 °C for 28 days, confirming its excellent specificity, reproducibility, and operational stability.

Yuwu Chi et al. [93] synthesized negatively charged gold nanoparticles and positively charged tris(bipyridine)ruthenium(II)-ion electrostatic nanoclusters (i.e., (-)-AuNPs/[Ru(bpy)_3_]^2+^ electrostatic nanoclusters). Each [Ru(bpy)_3_]^2+^ complex ion can be used as a SERS marker with a signal amplification function as it carries three bipyridine ligands. After combining the [Ru(bpy)_3_]^2+^ complex ion with the immunochromatographic test strip (ICTS) it can achieve the quantitative detection of cTnI in human serum within 5 min, and it has an LOD of 60 pg/mL. This will help in the discovery and use of novel SERS markers based on high-valence metal–multiligand complexes like [Ru(bpy)_3_]^2+^.

### 2.4. Surface Plasmon Resonance (SPR)

Rajeev K. Sinha [94] developed a low-cost SPR device based on wavelength modulation and an SPR sensor chip. The sensor chip is formed of a mixed self-assembled monolayer (SAM) of 1-octanethiol and 11-mercaptoundecanoic acid on an annealed gold thin film. Furthermore, SAM is treated with 1-ethyl-3-(3-dimethylaminopropyl) carbodiimide hydrochloride (EDC) and N-hydroxysuccinimide (NHS) to immobilize the cTnI monoclonal antibody. When this device and sensor chip are used to detect the cardiac biomarker protein cTnI then the LOD becomes as low as 0.03125 ng/mL.

**Figure 4:**
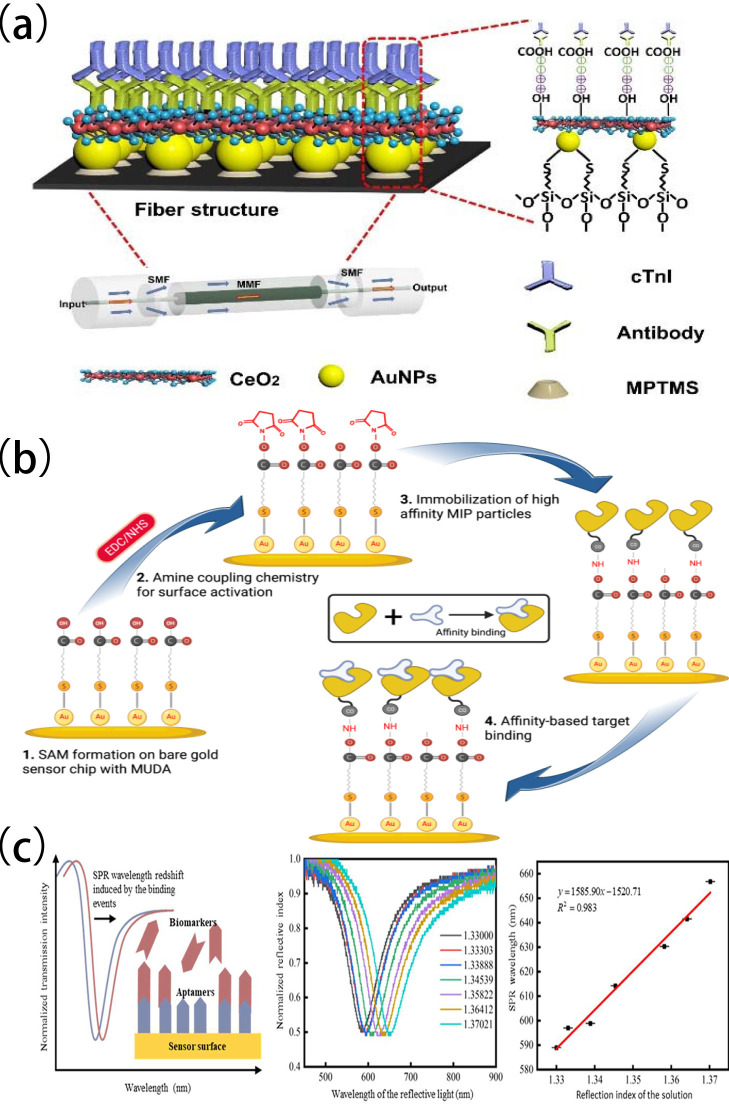
(**a**) The measurement principle of the fiber-optic-based SPR biosensor and its normalized spectra in solutions with different refractive indices; (**b**) the construction of the LSPR-based heterocore optical fiber sensor; (**c**) the schematic diagram of the bionic sensor constructed based on the portable SPR and epitope-imprinted synthetic receptors. (**a**) reproduced from Ref. [95], Copyright 2022, MDPI. (**b**) reproduced from Ref. [96], Copyright 2023, IEEE. (**c**) reproduced from Ref. [96], Copyright 2023, MDPI.

Ridong Wang [95] developed a fiber-optic-based SPR biosensor modified with a DNA aptamer specific for cTnI. The surface distribution of the aptamer can be precisely controlled by optimizing the surface concentration of the DNA aptamer and introducing a programmable DNA framework into the SPR biosensor (Figure 4a). Upon detection of cTnI the LOD of this sensor can reach 2.5 nM (57.5 ng/mL). The study utilized various real clinical samples including different pathological states (such as AMI patients and healthy controls) for verification. The results showed that in the presence of coexisting biomarkers with similar structures, such as cTnT, the sensor exhibited extremely low cross-reactivity, demonstrating excellent selectivity for the target molecule. During repeated detections of different batches of clinical samples the signal fluctuations were maintained within a minimal range, reflecting stable reproducibility. Additionally, after long-term storage (such as at 4 °C for a certain period) the sensor still maintained consistent signal output, confirming its good operational stability.

Yu Wang et al. [96] constructed a heterocore structure sensor based on localized surface plasmon resonance (LSPR) (Figure 4b). The performance, stability, and biocompatibility of the sensor probe can be improved by immobilizing gold nanoparticles (AuNPs) and cerium oxide nanoparticles (CeO_2_-NPs) in the sensing region. When used for detecting cTnI, the LOD and sensitivity of the constructed sensor are 108.15 ng/mL and 3 ng/mL. The sensor exhibited a detection limit (LOD) of 108.15 ng/mL and a high sensitivity of 3 ng/mL for cTnI in clinical matrices such as serum and plasma. During the study not only were calibration solutions used for quantitative calibration, but real clinical samples, including different pathological states (covering various biological samples such as chest pain patients and healthy controls), were also introduced to validate the detection performance, ensuring results were close to actual diagnostic scenarios. Performance evaluation showed that the sensor had excellent selectivity and no significant cross-reactivity with common interfering substances such as myoglobin and C-reactive protein. Meanwhile, it maintained good reproducibility in repeated tests (intra-batch coefficient of variation < 5%) and stable signal output after storage at 4 °C for 4 weeks. Its comprehensive performance provides a reliable technical support for the early and accurate diagnosis of AMI.

Zeynep Altintas et al. [97] used portable angular SPR and epitope-imprinted synthetic receptors to develop a bionic sensor for detecting cTnI (Figure 4c), which can yield quantitative detection of cTnI within a concentration range of 0.78–50 ng/mL and with an LOD of 0.52 ng/mL. The selectivity study compared to the control nanomolecularly imprinted polymer revealed that the binding affinity of the target nanomolecularly imprinted polymer for cTnI protein is 12 times higher. The specificity of the sensor was established by studying reference molecules, and the non-specific binding of interferents was within the range of 3–10%. This shows that this SPR sensor is fast, low-cost, and label-free.

### 2.5. Fiber-Optic Sensing Technology (FOST)

Yang Ran et al. [98] developed a highly integrated, portable, and sensitive fiber-optic immunosensor for cTnI using a functionalized phase-shifted micro-fiber Bragg grating (μFBG) probe. The principle involves regulating phase shifts to generate accurate reflection signals and improving spectral resolution to detect ultra-small refractive index changes caused by cTnI antigen capture for quantitative analysis. This sensor has a detection range of 0.1–10 ng/mL for cTnI, an LOD of 0.03 ng/mL, and excellent specificity, allowing detection in human serum samples. It also demonstrates strong competitiveness in the field of AMI point-of-care testing (POCT).

Li Yang et al. [99] designed an all-optical chemiluminescence collection cell (CC cell) to address the low collection efficiency of chemiluminescence emission in microscale spaces during total internal reflection in optical fibers. The CC cell features a concave mirror at the bottom and a coaxial tubular mirror on the wall, removing the need for complex equipment and other chemical reagents. Testing with cTnI as a cardiac biomarker demonstrated accurate detection across a linear range of 1–80,000 pg/mL, with a low LOD of 0.31 pg/mL—two orders of magnitude lower than that of conventional chemiluminescent fiber sensors—significantly improving detection sensitivity.

**Figure 5:**
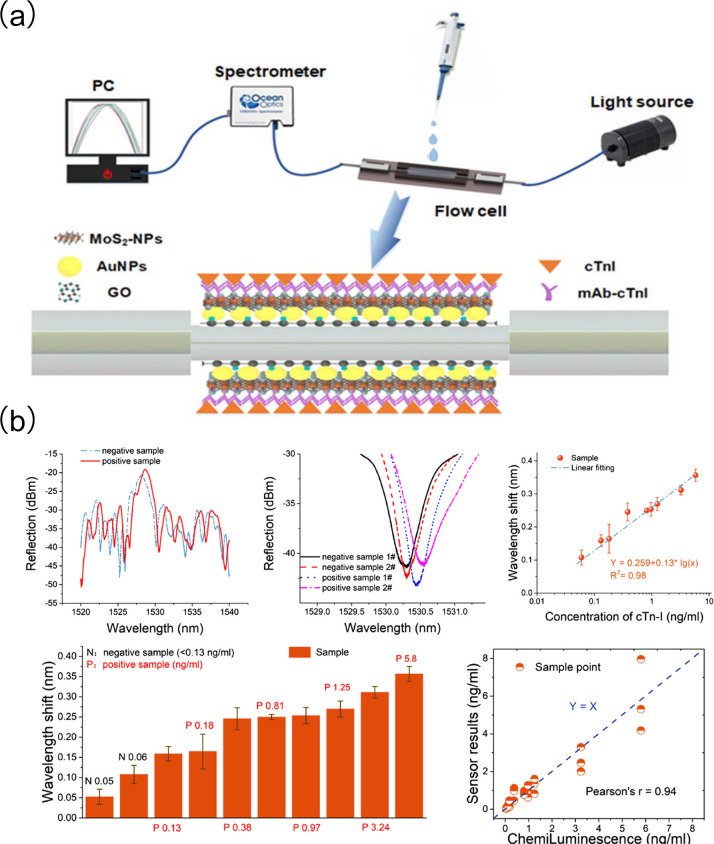
(**a**) Fiber-optic biosensor based on LSPR technology; (**b**) μFBG biosensor modified with antibodies and 50-fold diluted gold, showing response plots during the detection of a series of clinical serum samples. (**a**) reproduced from Ref. [100], Copyright 2022, IEEE. (**b**) reproduced from Ref. [101], Copyright 2023, ScienceDirect.

Santosh Kumar et al. [100] developed a fiber-optic biosensor for cTnI detection using localized surface plasmon resonance (LSPR) technology, and an etched multi-mode-photosensitive-multi-mode (MPM) fiber structure with a core mismatch (Figure 5a). The etched MPM fiber surface was functionalized with graphene oxide (GO), gold nanoparticles (AuNPs), and molybdenum disulfide nanoparticles (MoS_2_-NPs) to improve the sensitivity and stability of the sensor probe. Enzymatic functionalization of the sensing surface further improved selectivity. Through the directional anchoring of specific recognition molecules, cross-reactivity with interfering substances such as myoglobin and C-reactive protein was significantly reduced, enhancing its selectivity. The sensor demonstrated good detection performance in clinically relevant serum matrices, exhibiting a linear range of 0–1000 ng/mL, an LOD of 96.2638 ng/mL, and a sensitivity of 3.4 pm/(ng/mL). To validate its practical utility, the study employed not only calibration solutions for quantitative calibration but also diverse real-world clinical samples from acute chest pain patients, coronary artery disease patients, and healthy individuals. Results showed that the sensor maintained an inter-batch coefficient of variation as low as 7% across different detection runs, demonstrating reliable reproducibility. Moreover, after 28 days of storage at room temperature the detection signal attenuation rate was less than 10%, highlighting its remarkable stability.

Jun Guo et al. [101] developed a conical needle-shaped micro-fiber Bragg grating (μFBG) sensor surface modified with plasmonic gold nanostars (AuNSts). The star-shaped nano-tips of AuNSts improve the electromagnetic field, significantly improving sensor sensitivity, whereas electrostatic attraction accelerates detection kinetics. This sensor responds to cTnI concentrations below 10 ng/mL, with an LOD of 3 pg/mL in buffer and 11.9 pg/mL in clinical serum, and completes detection within approximately 5 min (Figure 5b). During the study not only were calibration solutions used for basic performance calibration, but clinical serum samples (including real biological samples from acute myocardial injury patients and healthy controls) were also employed to validate the actual detection performance. Through specific molecular modification on the surface of gold nanostars the sensor exhibited high selectivity for troponin I, with a cross-reactivity rate of less than 2% against common interfering substances such as myoglobin and C-reactive protein. In reproducibility tests the signal fluctuation range across different batches was less than 5%, demonstrating good detection consistency. Additionally, after 21 days of storage at 4 °C the detection signal attenuation rate was only 8%, indicating stable long-term performance.

### 2.6. Photoelectrochemical Sensing Technology (PEC)

**Figure 6:**
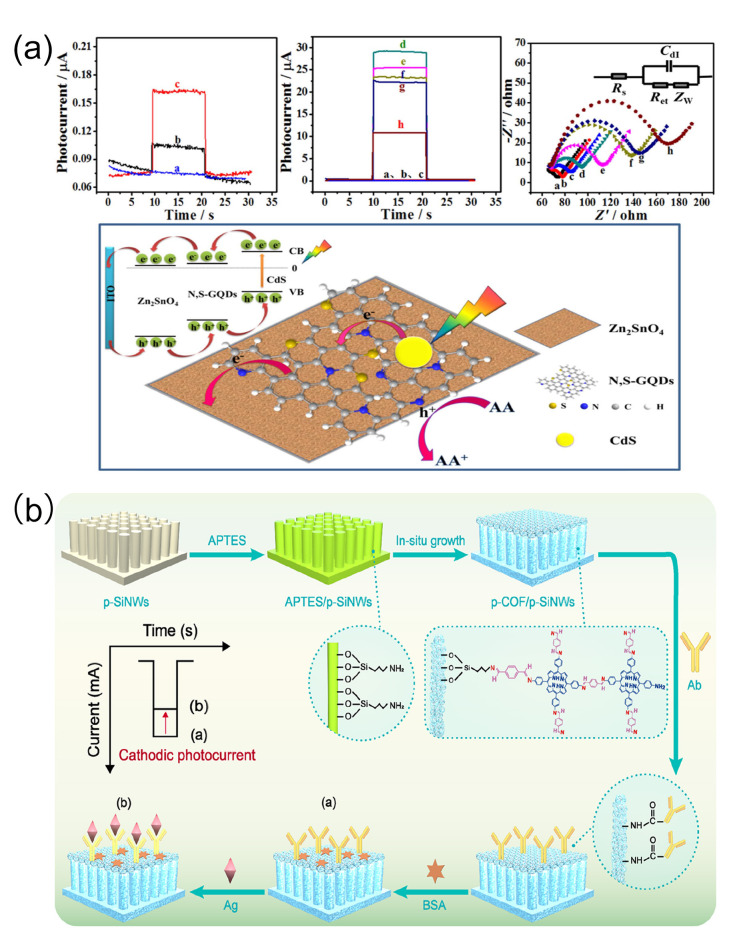
(**a**) Schematic illustration of the construction, photocurrent response, and electrochemical impedance spectroscopy (EIS) plots of the label-free PEC sensor; (**b**) schematic diagram of the construction of the p-COF@p-SiNWs photocathode immunosensor and its recognition of cTnI. (**a**) reproduced from Ref. [102], Copyright 2018, ScienceDirect. (**b**) reproduced from Ref. [103], Copyright 2018, ScienceDirect.

Dawei Fan et al. [102] first prepared nitrogen and sulfur co-doped graphene quantum dots (N, S-GQDs) and CdS-sensitized hierarchical cubic Zn_2_SnO_4_, and then modified CdS nanoparticles via in situ growth to obtain a Zn_2_SnO_4_/N, S-GQDs/CdS composite with significant photocurrents. The label-free photoelectrochemical (PEC) sensor developed based on this composite (Figure 6a) had a photocurrent 30 times higher than that of pure cubic Zn_2_SnO_4_ when detecting cTnI. The photocurrents showed a linear decrease with the logarithm of cTnI concentration in the concentration range of 0.001 ng/mL to 50 ng/mL, with an LOD of 0.3 pg/mL. Researchers not only used calibration solutions to precisely calibrate the sensor’s performance but also collected various real clinical samples from acute myocardial infarction patients, other cardiac disease patients, and healthy individuals to comprehensively validate the sensor’s actual detection efficacy. Benefiting from the unique structure and properties of the composite material, the sensor exhibited high selectivity for cTnI, with almost no cross-reactivity toward common interfering substances such as myoglobin and C-reactive protein. In reproducibility tests, repeated detections of cTnI samples at the same concentration showed minimal differences in photocurrent data, ensuring reliable and consistent detection results.

Ding Wang et al. [103] developed a unique S-type heterojunction based on porphyrin-based covalent organic frameworks (p-COFs) and p-type silicon nanowire arrays (p-SiNWs), successfully developing a p-COF@p-SiNWs photocathode immunosensor for cTnI recognition. Here, p-SiNWs act as the photocathode platform to produce strong photocurrent responses, whereas p-COFs catalyze charge carrier migration through suitable band alignment with p-SiNWs. Their crystalline π-conjugated networks and abundant amino groups promote electron transfer and anti-cTnI immobilization. This sensor has a detection range of 5 pg/mL to 10 ng/mL in clinical serum samples, with a low LOD of 1.36 pg/mL (Figure 6b). Unlike commercial ELISA the RSD is 0.06–0.18% and the recovery rate is 95.4–109.5%.

Pei Song et al. [104] developed a novel technique for highly sensitive PEC immunoassay by introducing a biomimetic MOF-derived nanozyme, hemin/bovine serum albumin@zeolitic imidazolate framework-8 (Hemin/BSA@ZIF-8), into the target biorecognition and sensing process. This catalyzes substrate oxidation to form insoluble precipitates, preventing electron transfer and causing a significant decrease in photocurrent. The constructed PEC sensor yields a wide linear range of 0.1 pg/mL to 100 ng/mL for cTnI detection, with an LOD as low as 11.9 fg/mL. This work provides novel insights into the design of biomimetic MOF nanozyme-mediated PEC immunoassays and a novel sensing strategy for accurate human biomarker analysis.

Yingzi Fu et al. [105] prepared bismuth-doped tin oxide and tin disulfide heterojunctions (Bi-SnOS) using a hydrothermal method to be used as a highly sensitive sensing platform. By using copper sulfide-coated gold nanoparticles (Au@CuS) as dual quenching probes they developed an excellent PEC sensor for cTnI detection using sandwich immunoassay. Under appropriate conditions the proposed biosensor displays high-performance detection of cTnI, with a range of 0.1 pg/mL to 5.0 ng/mL and a low LOD of 44.7 fg/mL. This approach of adjusting band structures through doped metal ions and the formation of heterojunctions is an effective strategy for developing sensing platforms to achieve accurate disease detection markers.

In summary, the optical detection technology system for cTnI has formed a diversified development landscape. Local and global researchers have conducted multidimensional explorations on these technologies, developing optical sensor systems based on different principles, such as labeled, label-free, and real-time monitoring types. These technologies have been broadly used in cTnI molecular recognition. Table 1 lists specific detection techniques and sensor performance. They are advancing cTnI detection toward miniaturization and intelligence by improving specificity and sensitivity.

In optical technologies, IFAL offers advantages such as high sensitivity, simple operation, and suitability for rapid detection. However, it suffers from issues including the susceptibility of fluorescence signals to interference and the potential impacts of labeling on antibody activity. ELISA is low cost and features a batch detection capability, making it suitable for clinical routine scenarios, but it has cumbersome steps and limited sensitivity. SERS possesses single-molecule detection capability and ultra-high sensitivity, yet it is constrained by the reproducibility of nanosubstrate preparation and instrument costs. SPR enables label-free and rapid detection, but it relies on expensive equipment and imposes strict requirements on the detection environment. FOST has miniaturization and anti-interference advantages, fitting well with point-of-care testing needs, but its signal intensity and stability require optimization. PEC has low background signals and low energy consumption, but it depends on the preparation of photoelectric materials and faces electrode stability issues. Along with ongoing advancements in nanomaterials and biosensing technologies, optical detection approaches are expected to play an important role in clinical precision diagnosis, early warning, and point-of-care testing, eventually becoming a core component of CVDs diagnostic technology systems.

## 3. Electrical Detection Methods

The electrical methods for cTnI detection are different and efficient; they primarily include electrochemical immunosensing technology (EIT), electrochemical aptamer detection technology (EADT), field-effect transistor technology (FET), and electrochemiluminescence immunoassay (ECLIA).

Of note, EIT relies on a specific binding principle of antigen–antibody, immobilizing cTnI antibodies on the electrode surface. The electrochemical signal at the electrode surface changes immediately when cTnI in the sample binds to these antibodies. Researchers achieve a quantitative analysis of cTnI by accurately detecting variations in current or potential. cTnI is widely used in clinical emergency settings due to its high sensitivity and rapid detection.

EADT uses specific recognition properties of aptamers for cTnI. Upon binding to cTnI, the aptamer undergoes a structural change, influencing the electrochemical properties of the electrode surface. This technology is simple to operate, with stable aptamers, effectively minimizing detection costs.

FET modifies the gate of the field-effect transistor with cTnI-specific recognition elements. The electrical properties of the transistor change when cTnI binds to these elements. With benefits in miniaturization and integration, cTnI holds promise for developing convenient portable detection devices.

Notably, ECLIA combines electrochemical and chemiluminescence technologies. Electrolysis causes luminescent substances on the electrode surface to generate light signals, and the binding process of cTnI with antibodies influences signal intensity. ECLIA is extensively used in clinical laboratory testing due to its extremely high sensitivity.

### 3.1. Electrochemical Immunosensing Technology

**Figure 7:**
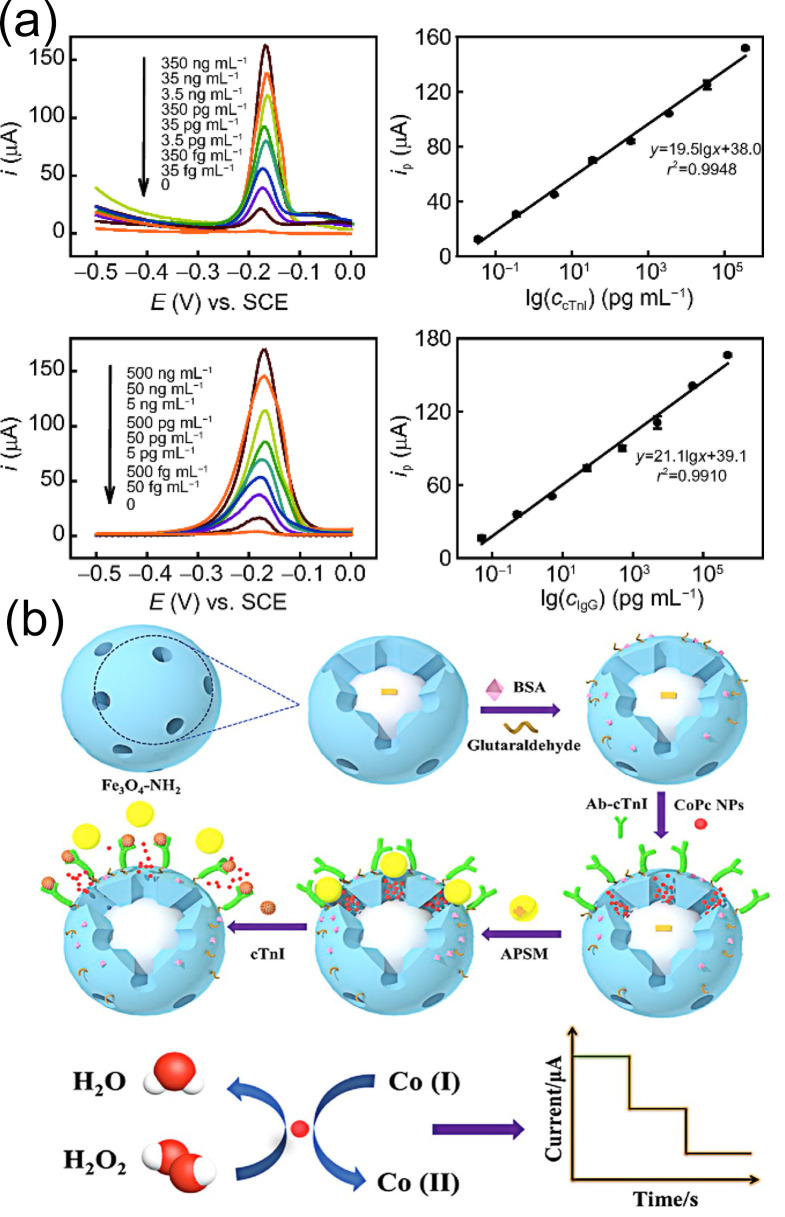
(**a**) Linear response plot of the Ab2-C-dots@AuNPs electrochemical immunosensor for cTnI detection; (**b**) schematic illustration of the preparation of an electrochemical immunosensor based on Fe_3_O_4_-NH_2_ and CoPc NPs. (**a**) reproduced from Ref. [109], Copyright 2018, Springer. (**b**) reproduced from Ref. [110], Copyright 2019, ScienceDirect.

Di Yang et al. [109] prepared hydroxyl-rich carbon dots-assisted gold nanoparticles (C-dots@AuNPs) and developed a sandwich-type immunosensor on a chitosan-modified glassy carbon electrode (GCE) via glutaraldehyde (GA) cross-linking. Carbon dots (C-dots) and C-dots@AuNPs catalyze the Cu^2+^ and ascorbic acid (AA) reaction, forming copper nanoparticles (CuNPs) that coat the surface of the original nanoparticles, hence amplifying the detection signal. The prepared electrode was applied to sandwich-type immunoassay using anodic stripping square wave voltammetry (ASSWV), reducing the LOD for cTnI to the fg/mL level (Figure 7a).

Dan Wu et al. [110] used aminated polystyrene microspheres (APSMs) as molecular gates and ferroferric oxide (Fe_3_O_4_) as nanocontainers. Amino-functionalized mesoporous ferroferric oxide (Fe_3_O_4_-NH_2_) was used to load cobalt phthalocyanine nanoparticles (CoPc NPs) and further capture the antibody (Ab) of cTnI, hence forming Fe_3_O_4_-Ab. A new antigen-responsive electrochemical immunosensor with a controlled-release system (Figure 7b) was designed for cTnI detection. Under optimal conditions, this sensor has a broad linear range from 1.0 pg/mL to 100 ng/mL and an LOD as low as 0.39 pg/mL. Additionally, through molecular gating interface modification the sensor exhibited high selectivity for cTnI, with cross-reactivity rates of less than 2% toward interfering substances such as myoglobin and C-reactive protein. Reproducibility tests showed that the intra-batch coefficient of variation (CV) for samples at the same concentration was less than 5% and the inter-batch CV was less than 8%, indicating good result consistency. After 4 weeks of storage at 4 °C the electrochemical signal attenuation rate of the sensor was only 7%, demonstrating stable long-term performance.

**Figure 8:**
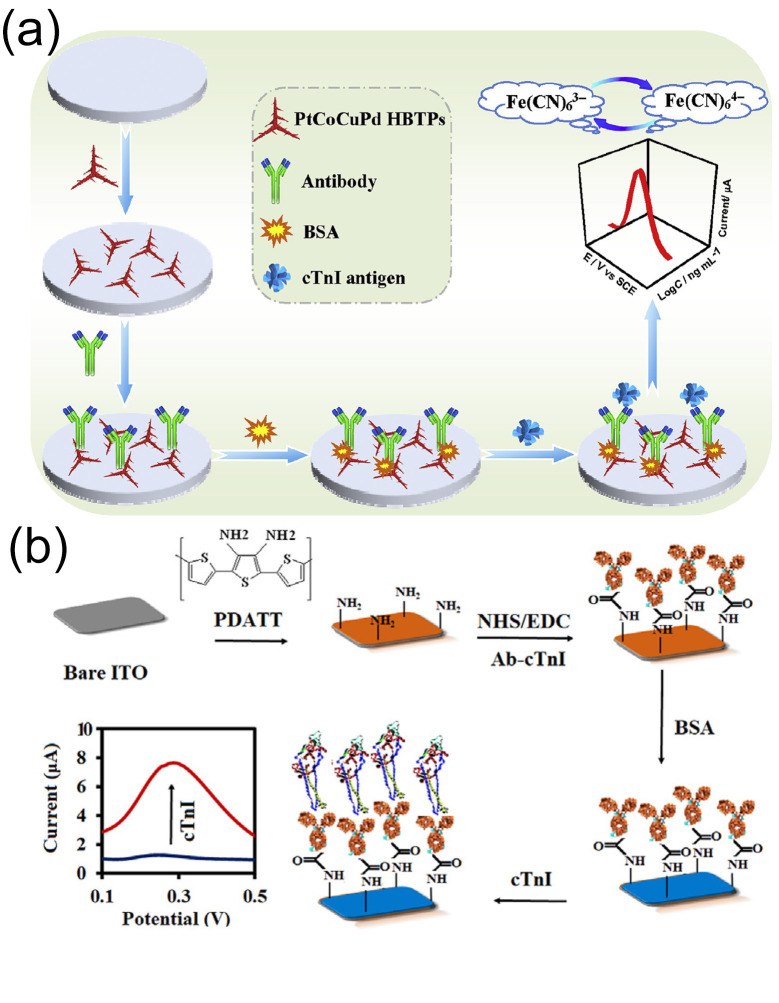
(**a**) Schematic illustration of the construction and application of a cTnI immunosensor based on PtCoCuPd hexagonal bipyramidal tetrahedral HBTPs and (**b**) the ITO/PDATT/cTnI-Ab/BSA electrochemical immunosensor for cTnI detection. (**a**) reproduced from Ref. [111], Copyright 2019, ScienceDirect. (**b**) reproduced from Ref. [113], Copyright 2021, ScienceDirect.

Jiu-Ju et al. [111] synthesized a quaternary-metal hierarchical branched tripodal structure (HBTP) composed of PtCoCuPd via a one-pot aqueous-phase method, notably performed without the use of seed crystals or organic solvents. This structure was used to construct a novel label-free immunosensor for detecting cTnI (Figure 8a). The unique hierarchical micro/nanostructure significantly enhances antibody immobilization and enhances the catalytic activity towards potassium ferricyanide (K_3_Fe(CN)_6_), effectively amplifying the electrochemical signal and improving the detection sensitivity. In human serum matrices the sensor demonstrated excellent detection performance for cTnI. It had a wide linear range of 0.001–100.0 ng/mL (spanning five orders of magnitude), with a detection limit (LOD) as low as 0.2 pg/mL. Validation using real clinical serum samples (including those from acute myocardial infarction patients and healthy controls) showed that the cross-reactivity to interferents such as myoglobin and CRP was <3.8%, the intra-batch/inter-batch RSDs were <4.2%, and the response signal of the electrode attenuated by <5.1% after storage at 4 °C for 30 days, confirming its high specificity, strong reproducibility, and long-term stability.

Li et al. [112] designed an electrochemical immunosensor based on a signal amplification strategy that integrates CDs-3D-PG-Pd@Au NCs with thionine (Th)-mediated H_2_O_2_ reduction. Notably, β-cyclodextrin (CD) improved the dispersibility of three-dimensional porous graphene (3D-PG) and exhibited a high affinity for the immobilization of Ab2. A substantial number of Pd@Au nanocubes, loaded onto the CDs-3D-PG, significantly enhanced the electrochemical signal, with the CDs-3D-PG-Pd@Au NCs composite serving as a signal enhancer. Under optimal conditions, the immunosensor showed excellent selectivity and reproducibility for cTnI detection, with an LOD as low as 33.3 fg/mL, indicating the potential of this approach for clinical applications.

O’Mullane et al. [113] fabricated a conductive film of poly(2,5-bis(2-thienyl)-3,4-diamino-terthiophene) (PDATT) on an indium tin oxide (ITO) electrode to develop a novel electrochemical immunosensor for selective affinity binding and the rapid detection of cTnI in plasma (Figure 8b). Differential pulse voltammetry (DPV) was used for detection upon formation of a cTnI immune complex on the sensor surface, achieving a response range of 0.01–100 ng/mL and an LOD as low as 0.01 ng/mL. The study validated the sensor using various clinical samples, including real plasma samples. The sensor exhibited high specificity for cTnI, with no significant fluctuations in response signals and a cross-reactivity rate of less than 5% in the presence of interfering substances (such as other serum proteins and myocardial marker analogs). The RSD of detection results for 0.1 ng/mL cTnI by five sensors prepared in the same batch was 3.2%. After storage at 4 °C for 21 days the sensor’s response signal to the standard solution remained above 92% of the initial value, indicating good selectivity, reproducibility, and long-term stability. This novel sensor is suitable for application in pathological laboratories and POCT for cTnI biomarker detection within 15 min.

Hashemnia et al. [114] constructed an electrochemical sensor for cTnI detection by utilizing tetrabromophenol blue (TBPB) as a chemical receptor, with the surface of a GCE modified with layered double-hydroxide nanostructures (LDHNS) and TBPB. The TBPB/mesoporous Fe/Co-LDHNS/GCE sensor, when operated in a pH 7.40 buffer containing 1 mM ascorbic acid (AA), exhibited cTnI detection via DPV, with a linear range of 50.00–3.50 × 10^5^ pM and an LOD of 2.77 pM.

### 3.2. Electrochemical Aptamer Detection Technology

Wong et al. [115] used mSiO_2_ along with an aptamer-based DNA nanostructure produced by hybridization chain reaction (aptHCR) to directly capture cTnI in clinical samples. The resulting nanocomplex was labeled with a custom DNA “turn-on” fluorescent dye (SPM). Enhanced fluorescence signals were detected using total internal reflection fluorescence microscopy (TIRFM), achieving an LOD as low as 8.5 fM with a high specificity for cTnI detection. This approach was suitable for emergency myocardial infarction diagnosis due to its inherent capabilities, including an optimal immunoreaction time of 30 min, lack of sample pretreatment, and the use of only 5 μL of serum.

**Figure 9:**
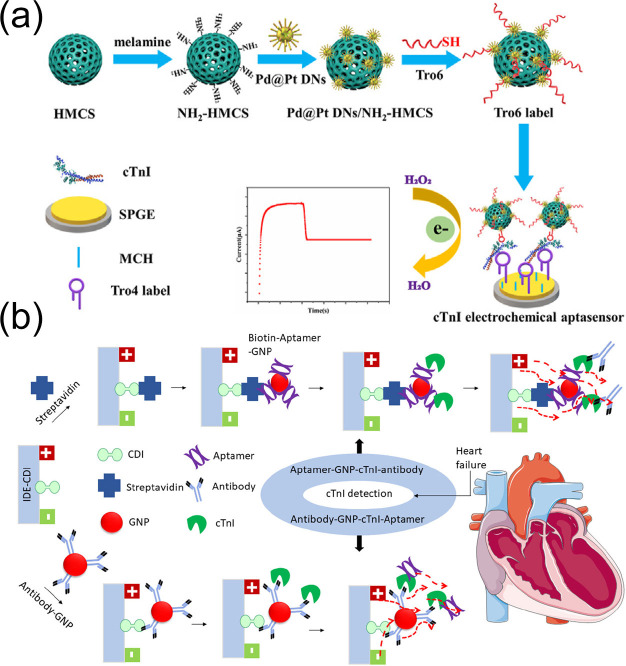
(**a**) Schematic diagram of the preparation of Pd@Pt DNs/NH_2_-HMCS/Tro6 markers. (**b**) Schematic diagram of the construction of a metal oxide interface immobilized with gold nanoparticles (AuNPs)-aptamer/antibody and its application for cTnI detection. (**a**) reproduced from Ref. [116], Copyright 2021, ScienceDirect. (**b**) reproduced from Ref. [59], Copyright 2021, ScienceDirect.

Zhao et al. [116] engineered a sandwich-type electrochemical aptamer sensor for cTnI detection using a screen-printed graphite electrode (SPGE) as the substrate for immobilizing capture aptamers. Core–shell Pd@Pt dendritic bimetallic nanoparticles supported on melamine-modified hollow mesoporous carbon spheres (Pd@PtDNs/NH_2_-HMCSs) were used as markers and were combined with thiol-modified DNA aptamer probes for signal amplification. This approach successfully developed an ultrasensitive sandwich-type electrochemical aptamer sensor for cTnI detection (Figure 9a). The detection was performed using human serum as the matrix, and the recovery was validated by standard addition method for spiked human serum samples. The linear range for cTnI detection was wide (0.1 pg/mL to 100.0 ng/mL), and the detection limit was as low as 15.4 fg/mL (S/N = 3). There was no obvious cross-reactivity with common interfering proteins such as bovine serum albumin, myoglobin, and C-reactive protein. After storage at 4 °C for 7 days, the response signal remained above 92% of the initial value. The RSD of five independently prepared sensors for detecting cTnI at the same concentration was 4.8%. The results showed that the sensor had excellent performance, remarkable selectivity, and good stability and reproducibility. This sensor was also efficacious in the detection of spiked human serum samples, indicating its potential widespread clinical application for cTnI detection for the diagnosis of AMI.

Gopinath et al. [59] constructed interdigitated electrode sensors with different surface interfaces for cTnI detection by utilizing both capture aptamer-conjugated gold nanoparticles and detection antibody probes via an alternating sandwich mode (Figure 9b). Experimental results showed that the LOD for cTnI was 1 fM when using antibodies and further improved to 100 aM when using the aptamer–gold–cTnI–antibody sandwich mode. Additionally, control experiments showed that the sensor exhibited high specificity for cTnI when faced with non-immune antibodies, cTnT, and probe-free systems, with current changes caused by cross-reactivity with interferents such as cTnT being less than 5%. It maintained stable responses to cTnI in spiked human serum containing 100-fold cTnT, demonstrating strong anti-contamination capability. After storage at 4 °C for 7 days the detection signal retention rate exceeded 92%. The RSD of five sensors from the same batch for detecting cTnI at the same concentration was 4.8%. The study not only optimized performance using calibration solutions but also validated them through spiking real human serum samples, with recovery rates ranging from 95% to 105%. The results indicated that the sensor had excellent performance, remarkable selectivity, and good stability and reproducibility, confirming its clinical utility. Combining the high adsorptivity of metal oxides with the signal amplification effect of gold nanoparticles, this sensor provides a highly sensitive and specific new scheme for the early diagnosis of acute myocardial infarction.

**Figure 10:**
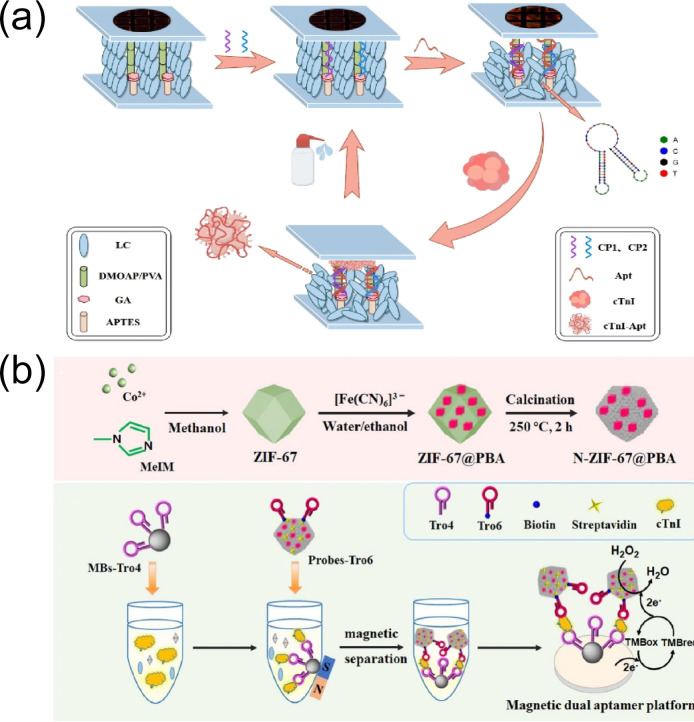
(**a**) Fabrication of the liquid crystal-based aptamer sensor and (**b**) schematic illustration of the N-ZIF-67@PBA sensor preparation and its application for cTnI detection. (**a**) reproduced from Ref. [117], Copyright 2021, American Chemical Society. (**b**) reproduced from Ref. [118], Copyright 2025, ScienceDirect.

Jin et al. [117] developed a label-free liquid crystal aptamer sensor for cTnI detection. This method leveraged nucleic acid hybridization and the specific binding between aptamers and target molecules (Figure 10a) using 3-aminopropyltriethoxysilane (APTES) and glutaraldehyde (GA). Notably, this method covalently linked CP1 and CP2 oligonucleotide chains complementary to the two ends of the aptamer to the substrate. The aptamer formed a π structure with CP1/CP2 via nucleic acid hybridization as a target capture probe. The study found that within the cTnI concentration range of 0.01–25 ng/mL the bright area coverage (Br) in the polarization microscope images of the sensor was linearly correlated with the logarithm of the cTnI concentration, with an LOD of 5.16 pg/mL. Using human serum as the detection matrix, spiked recovery tests were conducted on serum samples from healthy adult male subjects, yielding recovery rates of 97–102%, which validated the sensor’s practical applicability in real samples. Specificity tests showed that the sensor exhibited no significant response to non-target proteins such as immunoglobulin G, human serum albumin, myoglobin, high-sensitivity C-reactive protein, heart-type fatty acid-binding protein, and troponin T, demonstrating high selectivity and low cross-reactivity. The RSD for detecting the same cTnI concentration using sensors prepared in the same batch was less than 5%, indicating good reproducibility. Moreover, the sensor demonstrated stable performance under optimized conditions, providing a new method for the rapid detection of cTnI.

Wang et al. [118] used a hydrothermal synthesis technique to engineer ZIF-67 material with a high specific surface area. Subsequently, a Prussian blue analog (PBA) was grown in situ on the surface of the material via an ion exchange process, resulting in the formation of a MOF-on-MOF heterostructure denoted as ZIF-67@PBA. The N-ZIF-67@PBA electrocatalyst was obtained through low-temperature calcination. Subsequently, using this catalyst as a signal amplifier and combining the efficient separation of magnetic beads (MBs) they constructed a magnetic dual-aptamer electrochemical sensor for cTnI detection (Figure 10b). The sensor exhibited a good linear relationship within the concentration range of 10 fg/mL to 1 ng/mL using human serum as the detection matrix, with a detection limit (LOD) as low as 0.31 fg/mL. The study validated spiked recoveries using real human serum samples, achieving recovery rates of 98.5–100.4% and RSDs of 0.6–4.0%, which confirmed its practicality in complex biological matrices. Selectivity tests showed that when exposed to interferents such as human serum albumin, prostate-specific antigen, and thrombin (at concentrations 10 times that of the target) the sensor’s response signal to cTnI showed no significant fluctuations, with cross-reactivity rates below 5%. Five sensors prepared in the same batch exhibited an RSD of 1.1% for detecting the same cTnI concentration, demonstrating excellent reproducibility. After storage at 4 °C for one week, the sensor’s current signal retained 91.4% of its initial value, indicating good stability. These results suggested that MOF materials hold considerable potential for applications in the field of electrochemical aptasensing.

Sun et al. [119] designed a nanoporous electrochemical aptamer (E-AB) sensor for the rapid and sensitive detection of cTnI in a complex body fluids-based truncated aptamers approach. The electrochemical alloying/dealloying technology was used to prepare nanoporous electrodes, thereby increasing the active area of the sensor, which facilitated the specific detection of cTnI in serum and blood samples. The sensor achieved an LOD of 1 pg/mL, excellent stability, and high selectivity, indicating its superior potential for clinical application as a potent tool for rapid detection of AMI.

### 3.3. Field-Effect Transistor Technology (FET)

**Figure 11:**
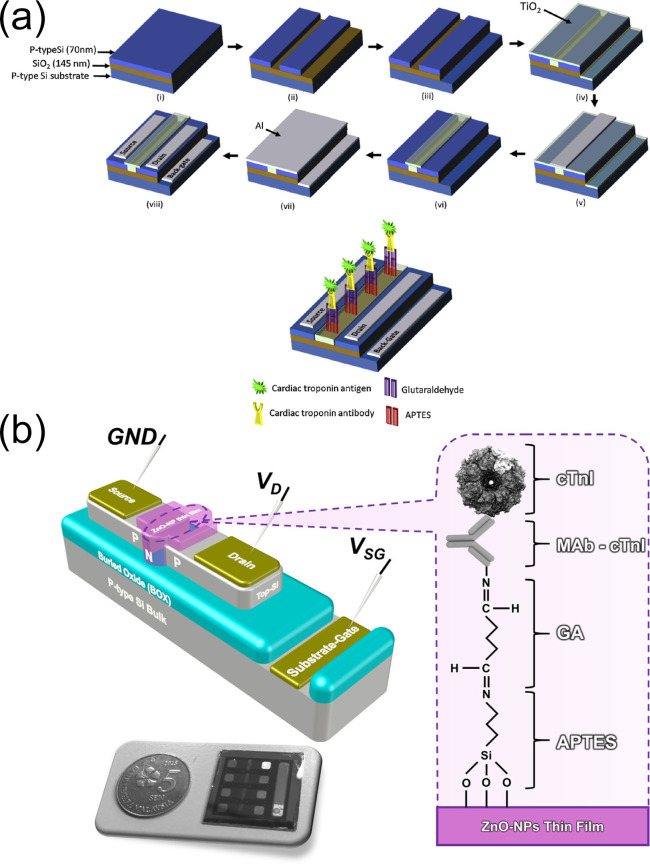
(**a**) Fabrication process of back-gate coupling for field-effect transistor (FET)-based biosensors and (**b**) schematic illustration of the preparation of ZnO-FET biosensor and its application for cTnI detection. (**a**) reproduced from Ref. [120], Copyright 2017, ScienceDirect. (**b**) reproduced from Ref. [121], Copyright 2017, ScienceDirect.

Arshad et al. [120] developed a label-free back-gate field-effect transistor (FET) device integrated with p-type anatase TiO_2_ on the sensing surface. Given that the TiO_2_ film exhibits superior performance in surface functionalization and electrical modulation, optimal cTnI detection was observed to be in the concentration range from 1 ng/mL to 10 μg/mL, with an LOD of 0.238 ng/mL. Additionally, the sensitivity of the device for cTnI detection increased to 2.438 μA/(g/mL). Studies showed that the device exhibited high selectivity for cTnI, with no significant response to common interfering proteins in human serum such as immunoglobulin G and human serum albumin, and the cross-reactivity rate was below 5%. Five sensors prepared in the same batch showed an RSD of 4.8% for detecting the same concentration of cTnI, demonstrating good reproducibility. After storage at 4 °C for one week the current signal of the device remained above 91% of the initial value, indicating excellent stability. Notably, with the integration and optimization of electronic devices this method holds significant potential for clinical diagnosis of various diseases, including cancer (Figure 11a).

Arshad et al. [121] fabricated a substrate-gated FET by depositing zinc oxide nanoparticle (ZnO-NP) films onto the channel region via a combination of sol–gel synthesis and spin-coating techniques, enabling effective integration of the sensing layer with the device architecture (Figure 11b). Subsequently, monoclonal antibodies against cTnI (MAb-cTnI) were then covalently immobilized on the ZnO-NPs film surface, and upon interaction with cTnI targets significant changes in drain current and threshold voltage occurred. This sensor demonstrated an LOD as low as 3.24 pg/mL for cTnI detection. The detection was conducted using human serum as the matrix. In the study, real human serum samples were used for spiked validation, with recovery rates ranging from 98.5% to 102% and with RSDs of 0.6–4.0%. The sensor showed minimal response to interferents such as troponin T and human serum albumin, with cross-reactivity rates below 5%, and only produced significant current changes in response to cTnI. Five sensors prepared in the same batch had an RSD of 1.9% for detecting the same concentration of cTnI. After storage at 4 °C for one week the device’s current signal remained above 93% of the initial value, indicating that the sensor possessed good specificity, excellent reproducibility, and outstanding stability. This approach paves the way for the future development of novel FET biosensors using advanced nanomaterials.

Wang et al. [122] developed an extended-gate EDL-gated FET biosensor, characterized with the ability to overcome electrostatic shielding. Additionally, this biosensor facilitates “ready-to-use” screening of target proteins with a single drop of blood in 5 min. This FET sensor demonstrates potent utility for early detection of cTnI in both hospital and home settings.

Pan et al. [123] prepared an extended-gate field-effect transistor (EGFET) pH sensor for cTnI detection in patient serum by depositing a titanium nitride (TiN) sensitive film as a sensing layer on an n^+^-type silicon substrate via DC sputtering. Carboxyl-terminated cTnI antibodies were activated using 1-ethyl-3-(3-dimethylaminopropyl) carbodiimide (EDC) and N-hydroxysuccinimide (NHS) and subsequently immobilized on the APTES-functionalized TiN sensitive film. The cTnI EGFET biosensor exhibited a high sensitivity of 21.88 mV/pC cTnI within a linear detection range of 0.01–100 ng/mL. These characteristics are comparable to results with a commercial ELISA kit.

### 3.4. Electrochemiluminescence Immunoassay (ECLIA)

Lu et al. [124] designed a dual-wavelength ratiometric ECL biosensor for cTnI detection. Gold nanoparticle-modified graphitic carbon nitride nanosheet composites (Au-CNNs) were prepared and used as donors, characterized by a stable ECL signal at 455 nm, which is thereby highly compatible with the absorption spectrum of Au nanoparticle-loaded graphene oxide/polyethylenimine (GPRu-Au), constructing an efficient electrochemiluminescence resonance energy transfer (ECL-RET) sensing platform. The platform showed high sensitivity for cTnI, with a detection range of 10 fg/mL–10 ng/mL and an LOD as low as 3.94 fg/mL.

**Figure 12:**
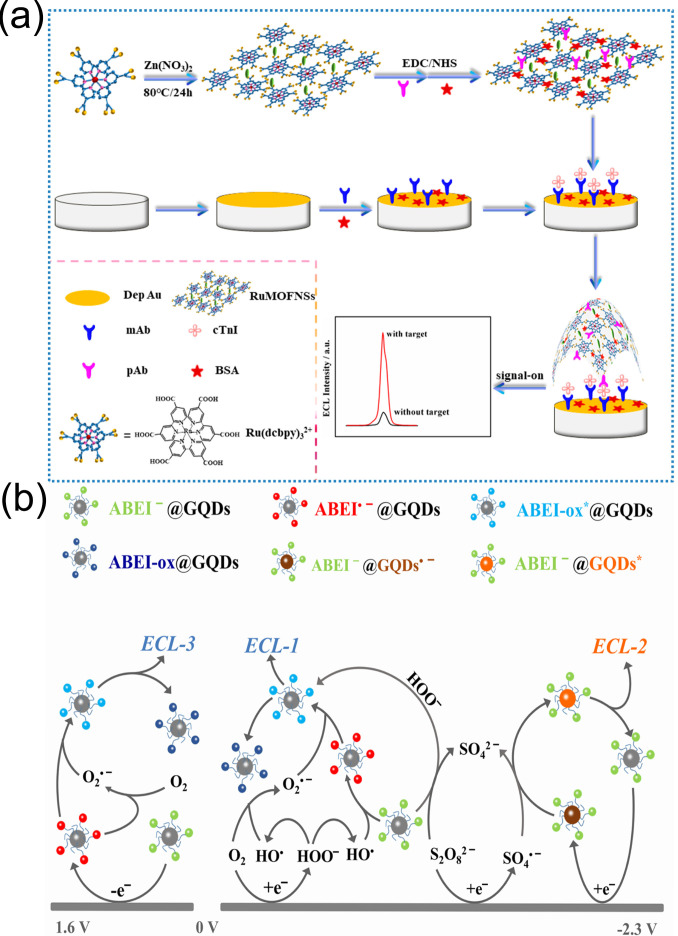
(**a**) Fabrication of the RuMOFNSs-Ab1/cTnI/BSA/mAb/depAu/GCE electrochemiluminescence immunosensor and (**b**) schematic illustration of cTnI recognition using ECL from ABEI@GQDs nanoluminophores with K_2_S_2_O_8_ as the co-reagent. (**a**) reproduced from Ref. [125], Copyright 2019, American Chemical Society. (**b**) reproduced from Ref. [126], Copyright 2021, ScienceDirect.

Yang et al. [125] used carboxyl tris(4,4′-dicarboxy-2,2′-bipyridyl)ruthenium(II) (${Ru(dcbpy)}_{3}^{2+}$) as an organic ligand to synthesize two-dimensional metal–organic framework (MOF) nanosheets (RuMOFNSs) with excellent ECL performance using a one-pot method. This approach facilitated the construction of a “signal-on” ECL immunosensor for cTnI detection (Figure 12a). The immunosensor showed high sensitivity and selectivity for cTnI, with a detection range of 1 fg/mL-10 ng/mL and an LOD as low as 0.48 fg/mL. Studies employed real human serum samples for spike validation, with recovery rates ranging from 95.14% to 104.75% and RSDs of 1.44–3.11%. The sensor showed weak responses to interferents such as troponin C, troponin T, and human serum albumin, with cross-reactivity rates below 5%. After 11 consecutive cyclic scans the RSD values for detecting 100 pg/mL and 1 fg/mL cTnI were 3.06% and 2.11%, respectively. Six sensors prepared in the same batch exhibited an RSD of 2.56% for detecting 1 ng/mL cTnI. The research indicated that the sensor possessed specific selectivity, good stability, and excellent reproducibility, providing a new strategy for the ultrasensitive detection of cTnI in clinical samples.

Shu et al. [126] synthesized a nanoluminophore consisting of N-(4-aminobutyl)-N-ethylisoluminol-functionalized graphene quantum dots (ABEI@GQDs), which exhibited three well-resolved, dual-color ECL emissions upon reaction with K_2_S_2_O_8_ as a co-reactant in aqueous solution. Through layer-by-layer assembling of ABEI@GQDs and antibodies on a chitosan-modified fluorine-doped tin oxide electrode they constructed a label-free three-potential ratiometric ECL immunosensor for cTnI detection (Figure 12b). This biosensor exhibited a linear detection range of 1.0 fg/mL–5.0 pg/mL for cTnI with an LOD of 0.35 fg/mL, outperforming most of the existing ECL methods. Selectivity studies showed that when the concentrations of human-derived proteins such as myoglobin (Mb), heart-type fatty acid-binding protein (hFABP), and BSA were 10 times that of cTnI, and the sensor’s ECL response values (I_2_/I_1_/I_3_) for cTnI were significantly higher than those for the interferents, with extremely low cross-reactivity. In terms of stability the ECL intensity of ABEI@GQDs nanoluminophores showed no significant difference within one month, and the sensor exhibited good reproducibility with an intraday precision (RSD) of 5.68% and an interday precision of 7.21% for 0.1 pg/mL cTnI. The study also validated the sensor’s performance using real human serum samples, with spiked recovery rates of 91–109%. When compared with the results from a chemiluminescent immunoassay kit (CLIAK) the RSD was ≤7.04%, indicating its reliable detection capability in actual biological matrices.

Li et al. [127] constructed a novel ECL biosensor using Co_3_O_4_ nanoarrays (NAs) as the sensing substrate and graphene quantum dots (GQDs)-coupled gold nanoclusters (Au NCs) nanocomposites as the signal markers. The Au NCs synthesized with glutathione as a ligand exhibited stable ECL signals in triethylamine (TEA) aqueous solution. Integrating GQDs with the preceding components resulted in the construction of the quantum-state composite (Au NCs-GQDs) exhibiting close spatial correlation, thereby generating a stronger ECL response due to resonance energy transfers and synergistic effects. This biosensor detected cTnI with a linear detection range of 500 fg/mL–20 ng/mL and an LOD as low as 354.2 fg/mL.

Sun et al. [128] constructed a sensitive sandwich-type ECL immunosensor for cTnI detection. Notably, molybdenum disulfide@cuprous oxide–silver nanoparticles (MoS_2_@Cu_2_O-Ag) were used to immobilize cTnI capture antibodies (Ab1s), and cerium-doped zinc oxide@nitrogen-doped graphene quantum dots (Ce:ZnO@NGQDs) loaded signal antibodies (Ab2s). MoS_2_@Cu_2_O-Ag nanoparticles feature good conductivity, biocompatibility, and a large specific surface area. On the other hand, Ce:ZnO, a co-reaction promoter, improves electron exchange rates, thereby enhancing the ECL signal. Under optimal conditions, the sensor exhibited a linear detection range of 10 pg mL^−1^–100 ng mL^−1^ and an LOD of 2.90 fg/mL, achieving excellent performance for cTnI detection in serum samples which indicates its potential application in biomolecule analysis.

In summary, global concern for early diagnosis of CVDs has significantly led to heightened systematic explorations on electrochemical detection technologies for cTnI. This has contributed to the construction of various electrochemical sensing systems based on multiple principles such as amperometric, impedimetric, and potentiometric types (see Table 2). These advanced technologies have overcome the limitations of traditional detection methods in terms of sensitivity and detection range by incorporating nanomaterial modification (such as carbon nanotubes, metal–organic frameworks), optimization of biological recognition elements, and signal amplification strategies (such as enzyme catalysis and electrochemiluminescence coupling). As a result, these technologies have facilitated ultra-trace detection of cTnI, achieving detection limits down to the fg/mL level and supporting wide dynamic range analysis.

Electrochemical methods used for the detection of cTnI have their unique advantages in application while also having certain limitations. EIT has high sensitivity, low cost, and convenient operation, but it has problems such as strong antibody dependence, cross-reactivity risk, and difficulty in electrode regeneration; EADT has superior specificity, high stability, and convenient synthesis, but faces challenges such as time-consuming aptamer screening, limited signal intensity, and insufficient compatibility with complex samples; FET combines miniaturization, real-time dynamic monitoring, and high sensitivity, but is susceptible to environmental interference, has a complex preparation process, and has biocompatibility issues; ECLIA has ultra-high sensitivity, wide linear range, and high automation, but is limited for grassroots applications due to high reagent costs, strong equipment dependence, and background interference risks. With the integration of microfluidic chip technology and machine learning algorithms, electrochemical detection platforms are evolving toward miniaturization and intelligence. These novel techniques are expected to facilitate the development of real-time dynamic biomarker monitoring networks for early screening, postoperative monitoring, and personalized treatment of CVDs, thereby providing critical technical support for precision medicine.

## 4. Other Detection Methods

Innovative technologies for cTnI detection continue to enhance detection capacity in this area. Lateral flow immunoassay (LFIA) has garnered increasing attention in clinical rapid diagnosis due to its ease of operation and rapid detection capabilities. Notably, LFIA provides significant support for time-sensitive events such as in emergency care, facilitating quick delivery of results. Microfluidic technology (MFT) focuses on the precise manipulation of microscale fluids, enhancing both detection efficiency and accuracy through fine control of fluid flow in tiny channels. Subsequently, MFT holds significant potential for complex sample analysis, as well as both medical research and clinical practices requiring high accuracy. Intelligent sensing technology (IST) relies on advanced sensors and smart algorithms for intelligent signal perception and deep analysis, enabling sensitive capture and precise interpretation of cTnI-related signals, thereby providing an efficient and intelligent solution that facilitates precise and intelligent medical detection.

### 4.1. Lateral Flow Immunoassay (LFIA) Detection Technology

Kim et al. [132] developed a paper/polyvinyl alcohol (PVA) hybrid LFIA platform integrated with a smartphone and equipped with simple optomechanical components in the smartphone reader. This technique achieved human cTnI detection within 20 min with an LOD of 0.92 pg/mL and a coefficient of variation < 10%. Notably, the technique was low cost, given that the smartphone-based reader costs USD 9.42 per device and <USD 0.49 per test, but with results comparable to high-end instruments. Consequently, this approach offers valuable insights into the construction of paper-based diagnostic platforms for high-performance POCT.

Wu et al. [133] prepared ultra-stable and highly luminescent quantum dot microspheres@SiO_2_-COOH (QBs@SiO_2_-COOH) nanospheres by leveraging the microemulsion technology in conjunction with the dual-protection strategy. The nanospheres served as excellent probes in an LFIA platform, facilitating the construction of a novel LFIA nanosensor. This biosensor achieved rapid cTnI detection within 10 min with a 60 μL sample, achieving an LOD of 0.036 ng/mL and a detection range of 0.12–125 ng/mL, accompanied by good reproducibility and excellent stability.

Pan et al. [134] developed an LFIA sensor that utilizes the hierarchical dendritic metal films (HD–nanometals) and background fluorescence technology for cTnI detection. To enhance detection accuracy, they proposed an improved UNet++ network integrated with attention and residual modules to accurately segment fluorescence regions with varying intensities, especially for the weak signals. The correlation coefficient (R^2^) between the sensor features and cTnI concentration reached 0.994, validating the model’s optimal accuracy and reliability, thereby enhancing POCT accuracy and providing a robust framework for the development of novel fluorescence immunochromatography.

### 4.2. Microfluidic Technology (MFT)

**Figure 13:**
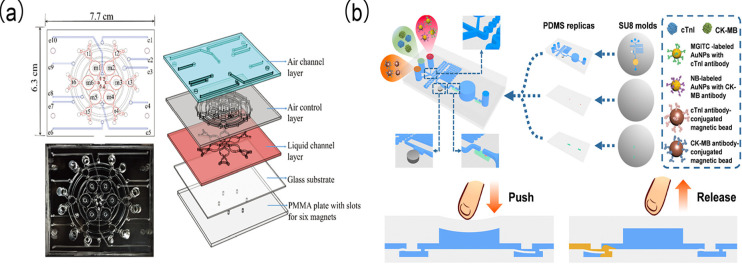
(**a**) Appearance and Design Schematic Diagram of the Microfluidic Chip; (**b**) schematic diagram of a SERS-based finger-pump microfluidic chip for simultaneous detection of two AMI biomarkers. (**a**) reproduced from Ref. [139], Copyright 2018, American Chemical Society. (**b**) reproduced from Ref. [138], Copyright 2023, ScienceDirect.

Lee et al. [135] constructed an enzyme-linked DNA aptamer-based detection method on an integrated microfluidic platform for sensitive and selective cTnI detection (Figure 13a). cTnI-specific aptamers were initially immobilized onto magnetic beads to capture proteins. These proteins subsequently bound to primary cTnI antibodies, followed by the attachment of horseradish peroxidase-labeled secondary antibodies. Quantitative detection using chemiluminescence intensity achieved an LOD of 12 ng/L with minimal off-target effects from other proteins.

Länge et al. [136] fabricated an electrochemical impedance spectroscopy (EIS) microfluidic chip composed of a microscope slide, sputtered electrodes, and polydimethylsiloxane (PDMS) microchannels which served as a label-free protein biosensor for cTnI detection. Notably, the application of a 1 ng/mL cTnI sample to the chip resulted in significant shifts in Nyquist plots, with both the sampling and measurement procedures conducted in minutes. This biosensor exhibits substantial potential for the new microfluidic impedance biosensor chips in the clinically relevant concentration detection of biomarkers.

Goel et al. [137] developed a flexible laser-induced graphene (LIG)-integrated microfluidic electrochemical biosensor, involving both flexible polymers polydimethylsiloxane (PDMS) and polyimide (PI) for microfluidic device fabrication. Biofunctionalized LIG electrodes prepared on PI substrates were combined with PDMS microchannels via modified thiol–epoxy click reactions. The developed biosensor, using a phosphate-buffered solution (PBS, pH 7.4) containing 0.1 M potassium chloride (KCl) as the matrix, exhibited good recognition ability for the quantitative detection of cTnI, with an LOD of 45.33 pg/mL. In the selectivity test the sensor’s response to cTnI was significantly higher than that to interfering proteins (such as myoglobin and bovine serum albumin) at 10-fold higher concentrations, showing extremely low cross-reactivity. In terms of stability, the LIG electrode maintained signal consistency after multiple detections, with intraday precision (RSD) below 8.5% and interday precision (RSD) below 10.2%, indicating good reproducibility. The study further validated the sensor using real human serum samples and subsequently the spiked recovery rates ranged from 89% to 105%, indicating that the sensor had reliable detection capabilities in complex biological matrices.

Gao et al. [138] prepared a single-channel finger-pump microfluidic chip integrated with surface-enhanced Raman scattering (SERS) and “sandwich” immunoassay technology, thereby facilitating highly sensitive cTnI detection within 5 min (Figure 13b). This microfluidic chip was able to achieve multiple reagent additions, remove unbound reactants, and eliminate the need for cumbersome syringe pumps. Additionally, this approach could be used to detect creatine kinase isoenzyme MB (CK-MB), another marker for AMI, enabling the simultaneous detection of two cardiac biomarkers in a single measurement. During the identification of cTnI and CK-MB, detection was performed using an aqueous solution containing phosphate-buffered saline (PBS, pH 7.4) as the matrix, and the LOD for both markers reached 0.01 ng/mL. In selectivity tests, the chip’s responses to cTnI and CK-MB were significantly higher than those to interfering proteins (such as myoglobin and bovine serum albumin) at 10-fold higher concentrations, exhibiting extremely low cross-reactivity. Stability experiments showed that the chip maintained good signal consistency across multiple repeated detections, with an intraday precision (RSD) below 7.8% and an interday precision (RSD) below 9.1% indicating excellent reproducibility. Further validation using real human serum samples yielded spiked recovery rates of 88–103%, and characteristic Raman signals remained stable in the complex serum matrix, suggesting reliable application potential in clinical samples. Therefore, the constructed single-channel finger-pump microfluidic chip was established as an effective tool for early AMI diagnosis.

### 4.3. Intelligent Sensing Technology (IST)

**Figure 14:**
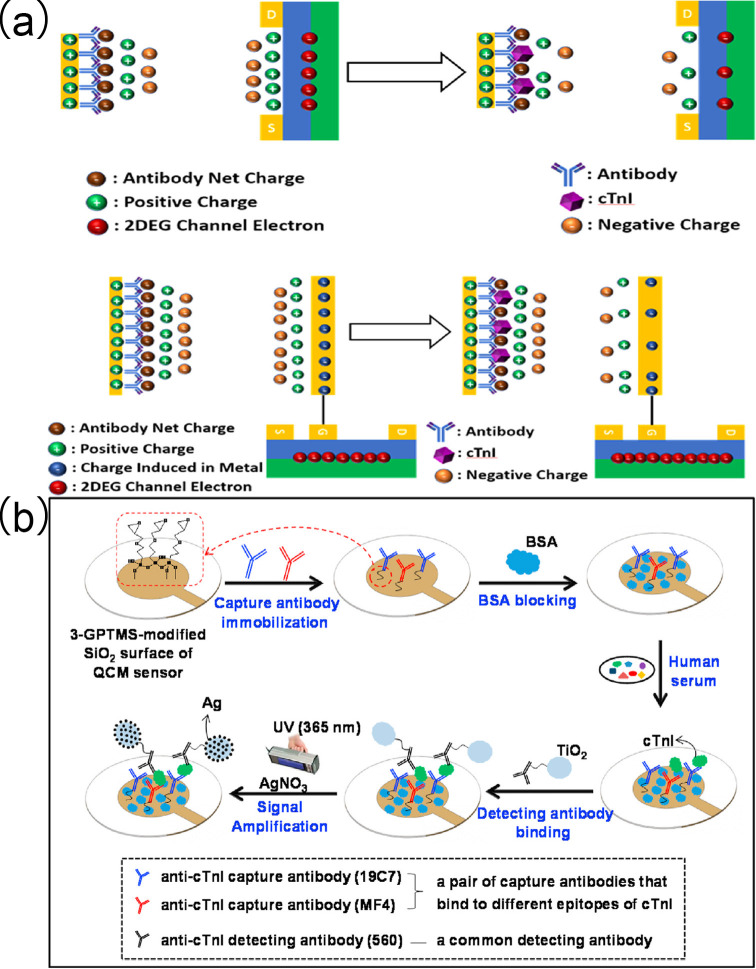
(**a**) Schematic illustration of charge and current changes caused by antigen–antibody binding in two types of sensors, and (**b**) schematic diagram of cTnI detection using a QCM biosensor chip incorporating TiO_2_-mediated photocatalytic silver staining signal amplification technology. (**a**) reproduced from Ref. [140], Copyright 2023, AIP Publishing. (**b**) reproduced from Ref. [141], Copyright 2021, ScienceDirect.

Ban [140] constructed a biosensor with ultrasensitive cTnI detection using two methods (Figure 14a). The first step involved the application of a pulsed bias voltage between the functionalized electrode and the gate of a double-layer high electron mobility transistor (HEMT), resulting in the charge changes correlated with cTnI concentration in the electrical double layer. The second method involved the fabrication of the sensing region on a glass slide, with pulsed gate signals externally connected to a nitride HEMT and thereby generating substantial charge accumulation changes for broader concentration range detection which is unaffected by charge shielding effects. Both designs exhibited excellent performance for cTnI detection, achieving an LOD as low as 0.01 ng/mL. The detection was performed using 1× phosphate-buffered saline (PBS, pH 7.4) containing 1% bovine serum albumin BSA as the matrix to simulate a physiological environment. In selectivity tests, the sensor exhibited significantly higher responses to cTnI than to interfering proteins (such as myoglobin and immunoglobulins) at 10-fold higher concentrations, with extremely low cross-reactivity. Stability assessments showed that the intraday precision (RSD) for multiple detections using the same device was below 6.8% and the interday precision (RSD) between different devices was below 8.3%, indicating good reproducibility. Although the study did not directly use real blood samples the sensor maintained high sensitivity in high-ionic-strength solutions (such as PBS with 1% BSA), suggesting applicability to complex biological matrices. This work provided a new direction for developing low-cost, portable chips for cTnI detection.

Lee et al. [141] prepared a highly sensitive and reproducible quartz crystal microbalance (QCM) immunosensor for cTnI detection in human serum. Signal amplification was achieved via photocatalytic silver staining, which increased the size of the TiO_2_ nanoparticle on the sensor surface. The QCM technique was employed to measure variations in resonance frequency due to mass changes on the sensor surface, enabling real-time monitoring of biomolecular interactions. A sandwich immunoassay format was used, followed by silver staining for mass enhancement, resulting in a significantly amplified signal. Under optimized conditions the sensor achieved an LOD of 18 pg/mL (Figure 14b), demonstrating improved sensitivity and assay reproducibility. This detection was carried out using a phosphate-buffered saline (PBS, pH 7.4) containing 1% bovine serum albumin as the matrix, and it was directly applied to the detection of cTnI in human serum samples. The dual-antibody design endowed the sensor with high selectivity. Even in the presence of interfering proteins such as myoglobin and immunoglobulin G at 10-fold higher concentrations in the serum, the response signal to cTnI was still significantly higher than those to the interferents. In terms of stability and reproducibility, the intraday precision (RSD) of the same sensor for 100 pg/mL cTnI was 10.2% and the interday precision (RSD) using three independent sensors on different days was 13%. Moreover, the standardized operation of the photocatalytic silver staining step ensured the consistency of signal amplification (the correlation coefficient R^2^ between the frequency change and the logarithm of cTnI concentration was 0.975). Blind spiked experiments showed that the recovery rates of cTnI in human serum samples by the sensor ranged from 81.4% to 82.8%. These results were significantly correlated with the results obtained by the clinical commercial VIDAS^®^ system (R^2^ = 0.998), confirming its reliable detection capability in real biological matrices.

Wang et al. [142] developed a portable biosensor for on-site cTnI detection by integrating the signal amplification function of a DNA walker with a low-cost glucometer (PGM). In the presence of cTnI, walker particles (WBs) undergo a rapid rolling motion along the surface of track particles (TBs), triggering the release of short DNA fragments conjugated with invertase. Invertase catalyzes hydrolysis of sucrose into glucose, which is then quantified by PGM. This “rolling walker-cTnI-glucometer’ (RWCG) strategy applied to clinical serum detection achieved an LOD of 0.001 ng/mL for cTnI within 8 s, which is suitable for on-site scenarios within emergency centers and homes. This biosensor exhibits significant clinical potential in the early diagnosis and treatment of cTnI-related conditions.

Ozcan et al. [143] developed a highly sensitive deep learning-enhanced paper-based vertical flow assay (hs-VFA) platform for cTnI detection. This approach involved the integration of innovations in amplified biosensing, imaging, and data processing. The biosensor achieved rapid and precise cTnI detection within 15 min per test with the use of 50 μL of serum. Leveraging gold-ion amplification chemistry and time-lapse imaging to enhance its performance, the platform achieved high sensitivity (LOD = 0.2 pg/mL) and accuracy (average coefficient of variation < 7%) for cTnI recognition. Notably, these findings indicate that this biosensor provides valuable reference for advancing research on the application of sensors for optimal outcomes in POCT sensors.

In summary, driven by the rising demand for precise diagnosis of CVDs, researchers have explored the application of intelligent technological innovations for cTnI detection. Consequently, a series of novel detection platforms with high sensitivity and portability have been developed through the integration of interdisciplinary insights (see Table 3). In the innovative techniques for detecting cTnI, LFIA, as a typical POCT technology, relies on its advantages of extremely simple operation, low cost, and rapid detection to become the preferred solution for grassroots healthcare and on-site screening. However, its detection limit typically only reaches the nanogram level, with insufficient quantitative capability and the risk of cross-reactivity. Conversely, MFT achieves high-throughput analysis and microliter-scale sample consumption by integrating multi-step processes such as sample processing and detection on a chip, matching the needs of miniaturized and automated detection. Nevertheless, its preparation relies on precision processing technologies, resulting in high mass-production costs, and its microchannels are prone to blockage by complex samples with delayed clinical translation. Furthermore, IST shows potential in femtomolar-level ultra-trace detection, real-time dynamic data analysis, and multi-modal anti-interference detection by means of nanomaterials or machine learning algorithms and can achieve remote interconnection through the Internet of Things. However, most of its core technologies are in the laboratory stage, with issues such as stability, biocompatibility, and data privacy needing urgent resolution, and it depends on high-end equipment support making widespread popularization difficult in the short term. Overall, LFIA is enhancing sensitivity through nanomaterials-based signal amplification techniques, MFT is focusing on preparation process optimization and standardization, and IST needs to break through technical maturity and cost bottlenecks, with the three jointly pushing cTnI detection toward the direction of convenience, precision, and intelligence.

These intelligent technologies facilitate ultrasensitive cTnI detection and the development of miniaturized (chip size ≤ 2 cm^2^), low cost (single-test consumable cost < $5), and POCT detection devices. Advances in flexible electronics and implantable sensing systems are anticipated to facilitate the development of comprehensive, full-cycle management systems for CVDs. The next-generation intelligent detection platforms aim to support early diagnosis, intraoperative monitoring, and postoperative rehabilitation by enabling real-time, dynamic biomarker monitoring, thereby promoting precision and personalized medicine.

## 5. Conclusions

Cardiovascular diseases remain the leading threat to global health, underscoring the urgent need for early diagnosis through precise biomarker monitoring. Notably, cTnI, a gold-standard biomarker for myocardial injury, plays a pivotal role in assessing the progression and clinical outcomes in patients with CVDs. Recent advancements in optical, electrical, and intelligent biosensors have significantly enhanced cTnI detection capabilities. However, these emerging technologies are characterized by persistent challenges in establishing a balance in their sensitivity, selectivity, and practicality for clinical translation.

Optical biosensors leverage fluorescence, surface plasmon resonance, or chemiluminescence to achieve ultrasensitive cTnI detection, with the LOD attaining ng/mL to pg/mL ranges. These emerging diagnostic systems leverage optimized nanomaterial-based signal amplification to enhance reproducibility, a critical factor for reliable early-stage clinical applications. Electrical biosensing platforms, including electrochemical and field-effect transistor platforms, demonstrate excellent performance in terms of rapid response times (typically < 15 min) and miniaturization, enabling POCT through the use of portable devices. Additionally, intelligent biosensors integrate microfluidics, nanotechnologies, and artificial intelligence (AI) models to automate sample processing and data analysis, thereby enhancing analytical throughput, diagnostic precision, and clinical utility.

However, standalone methods face inherent constraints. Optical sensors exhibit limited performance due to interference from ambient light, leading to signal instability. Additionally, electrical platforms exhibit the prevalence of nonspecific binding in complex biological matrices (such as serum whole-blood), compromising specificity. Furthermore, single-mode detection often fails to achieve a balance between high sensitivity and robust stability, thereby resulting in inconsistent outcomes in real-world clinical environments.

Hybrid strategies which integrate complementary technologies are emerging as transformative solutions. For instance, integrating electrochemical sensors with nanotechnology—such as gold nanoparticle-modified electrodes—enhances surface area and electron transfer kinetics, thereby achieving attomolar-level sensitivity. Also, integrating electrochemical detection with ELISA leverages antibody–antigen specificity to improve selectivity. Additionally, smartphone-integrated platforms enable real-time data processing, wireless transmission, and remote clinical consultation. Microfluidic systems streamline workflows by enabling direct cTnI detection in untreated samples (such as whole-blood), thereby minimizing the number of steps during preprocessing as well as the contamination risks.

Additionally, the rigor of the experimental designs needs to be strengthened. Currently, only some studies employ ≥ 20 blank measurements to evaluate the LOD and cross-matrix experiments (such as those using buffer, serum, and plasma) for validating the measurement range have not been widely adopted, indicating that the field’s emphasis on method reliability is still insufficient. The characterization of result reproducibility is inadequate as most papers do not provide inter-batch variation data and only present single experimental results, making it difficult to fully reflect the stability of the methods. The evaluation of matrix universality is lacking, with only a few studies reporting the ratio of LOD in serum to that in buffer. Consequently, this is leading to deviations in sensitivity comparisons across methods. The sufficiency and diversity of clinical samples are insufficient as sample sources are relatively homogeneous, which means they are failing to support the reliable evidence required for precise diagnosis.

To address these issues the following solutions are proposed: standardizing the LOD evaluation process; recommending the uniform use of ≥20 blank measurements; and validating the robustness of the measurement range through cross-matrix experiments (such as buffer, serum, and plasma). The latter consists of including intra-batch and inter-batch variation data (such as detection results from different batches of sensors) in result reporting to fully characterize method reproducibility; unifying the evaluation criteria for matrix universality, requiring the reporting of serum/buffer LOD ratios, and standardizing experimental conditions for sensitivity comparisons across methods; and expanding the inclusion of clinical samples to cover populations of different genders, ages, and disease states while also integrating clinical diagnostic gold standard data to enhance the clinical applicability of detection methods.

Substituting conventional antibodies with synthetic alternatives mitigates cost and stability limitations. Aptamers, engineered via the Systematic Evolution of Ligands by Exponential Enrichment (SELEX) technique, offer high affinity, thermal stability, and customizable modifications. Molecularly imprinted polymers (MIPs) function as synthetic recognition elements that emulate the specific binding characteristics of natural antibodies. These materials offer high chemical and thermal stability, cost-efficiency, and durability, making them well-suited for applications in challenging environmental conditions. These innovations enhance sensor longevity while lessening the dependency on cold-chain-dependent reagents.

Advances in flexible electronics and low-power microelectronics have led to the development of wearable cTnI sensors which are crucial for continuous CVDs monitoring. Smartwatches and epidermal patches incorporating electrochemical or optical modules hold the capacity to track cTnI fluctuations, thereby facilitating early intervention. These innovative technologies address the growing demand for decentralized healthcare, particularly in resource-limited settings.

Bridging the translational gap between laboratory research and clinical application necessitates robust interdisciplinary collaboration. Key priorities in this endeavor include the following: (1) the development of AI-driven predictive models for multi-biomarker correlation analysis; (2) comprehensive validation of sensor performance in large-scale clinical trials to establish standardized operating protocols; (3) exploration of CRISPR-based diagnostic platforms to achieve unparalleled specificity and sensitivity; and (4) enhancement of sensor antifouling properties using zwitterionic polymers or biomimetic coatings.

Contemporary cTnI detection technologies are evolving toward multiplexed, portable, and intelligent systems with the potential to revolutionize CVDs management. Through the integration of advanced material engineering, hybrid sensing modalities, and AI analytics, next-generation biosensors are poised to facilitate early diagnosis, personalized treatment, and improved patient prognoses globally.

This work systematically summarizes the optical, electrochemical, and intelligent detection methods for cTnI, deeply analyzes the performance characteristics and application bottlenecks of each technology, and proactively proposes optimization pathways for future detection strategies. In the field of optical detection, technologies such as IFAL and SERS have become research hotspots due to their high sensitivity advantages, but they generally face issues like fluorescence quenching and poor reproducibility in nanosubstrate preparation. For electrochemical detection methods such as ECLIA, whilst they offer portability and cost advantages they are also constrained by electrode stability and compatibility with complex samples. Emerging technologies represented by MFT and IST show potential in integration and intelligence but urgently need to address challenges such as insufficient technical maturity and high mass-production costs.

The study indicates that the scientific development of cTnI detection technologies in the future should focus on three major directions: cross-modal technology integration, such as constructing “optical-electrochemical” dual-signal amplification systems (e.g., combining SERS and ECLIA) to break through the sensitivity limits of single technologies through complementary advantages; biocompatible material innovation, developing new recognition elements like degradable nanocarriers and biomimetic aptamers to solve the environmental sensitivity of traditional antibodies/aptamers while reducing the biotoxicity of nanomaterials; and full-process intelligent integration, deeply coupling microfluidic chips with AI algorithms to achieve automated closed loops from sample injection and multi-index detection to risk prediction—for example, using machine learning models to dynamically analyze the correlation between cTnI concentration fluctuations and cardiovascular events. Additionally, the establishment of a standardization system (e.g., unifying detection thresholds and optimizing clinical validation processes) and the industrialization of portable devices (e.g., palm-top detectors integrated with IoT capabilities) will be key links in promoting technological transformation. Through these multi-dimensional innovations, future cTnI detection is expected to achieve synergistic improvements in sensitivity (femtomolar level), portability (POCT-oriented), and intelligence (risk prediction), providing more scientific technical support for early intervention and personalized treatment of cardiovascular diseases.

## Figures and Tables

**Table 1:** Comparison chart of optical detection methods for cTnI.

Techniques	Sensor	LOD (pg/mL)	Detection Range (ng/mL)	References
IFAL	Anti-cTnI/afGQDs immunofluorescence sensor	0.192	1 × 10^−3^–1 × 10^3^	[82]
	MOF@COU/Ab2 immunofluorescence sensor	0.099	1.998 × 10^−4^–0.6408	[83]
	MnO_2_-Ab@MQD immunofluorescence sensor	50	-	[85]
ELISA	ALP-triggered fluorescence ELISA	-	1–250	[87]
	ALP-induced immuno-sensor	30	0–175	[106]
	ALP-triggered fluorescence ELISA	50	2–150	[107]
SERS	SERS Sensor (AuNPs/GO/MB)	5	0.01–1000	[90]
	Magnetic molecularly imprinted SERS sensor (Fe_3_O_4_@ZrO_2_@Ag)	0.063	1 × 10^−3^–100	[91]
	SERS ratio-type aptamer sensor	0.27	1 × 10^−3^–100	[92]
	SERS immunochromatographic test strip ([Ru(bpy)_3_]^2+^ Electrostatic Aggregation)	60	-	[93]
SPR	SPR biosensor (Fe_3_O_4_@PDA-dAb/MWCNTs-PDA-AgNPs)	3750	-	[108]
	LSPR sensor (AuNPs/CeO_2_-NPs)	1.0815 × 10^5^	-	[96]
	Portable SPR biomimetic sensor	520	0.78–50	[97]
FOST	Optical fiber immunosensor	30	0.1–10	[98]
	All-optical chemiluminescence collection bottle	0.31	1 × 10^−3^–80	[99]
	Optical fiber biosensor (GO/AuNPs/MoS_2_-NPs)	96,263.8	0–1000	[100]
	Conical μFBG biosensor	3	<10	[101]
PEC	Unlabeled photoelectrochemical sensor (Zn_2_SnO_4_/N, S-GQDs/CdS)	0.3	1 × 10^−3^–50	[102]
	Photoanode immunosensor (p-COF@p-SiNWs)	1.36	5 × 10^−3^–10	[103]
	Biomimetic MOF nanozyme-mediated PEC sensor (Hemin/BSA@ZIF-8)	1.19 × 10^−2^	1 × 10^−4^–100	[104]
	Heterojunction PEC sensor (Bi-SnOS/Au@CuS)	4.47 × 10^−2^	1 × 10^−4^–5.0	[105]

**Table 2:** Comparison chart of electrical detection methods for cTnI.

Techniques	Sensor	LOD (pg/mL)	Detection Range (ng/mL)	References
Electrochemical Immunosensing Technology	Antigen-responsive electrochemical immunosensor (Fe_3_O_4_-Ab/CoPcNPs)	0.39	1 × 10^−3^–100	[110]
	Label-free electrochemical immunosensor (PtCoCuPd hierarchical dendritic tripod structure)	0.2	1 × 10^−3^–1 × 10^2^	[111]
	Conductive polymer film electrochemical immunosensor (PDATT/ITO)	10	0.01–100	[113]
	Electrochemical biosensor (Pd@PdPtCo MNPs)	0.031	1 × 10^−5^–200	[129]
Electrochemical aptamer detection technology	Sandwich-type electrochemical aptamer sensor (Pd@Pt DNs/NH_2_-HMCS)	0.0154	1 × 10^−4^–100	[116]
	Unlabeled electrochemical aptamer sensor (PPy-AuNPs modified SPCE)	25	0.05–0.5	[130]
	Liquid crystal aptamer sensor (nucleic acid hybridization and aptamer binding)	5.16	0.01–25	[117]
	Magnetic dual-aptamer sensor (MOF-on-MOF heterostructure ZIF-67@PBA)	3.1 × 10^−4^	1 × 10^−5^–1	[118]
FET	Unlabeled back-gate field-effect transistor (p-type anatase TiO_2_ film)	238	1–1 × 10^4^	[120]
	Substrate-gate + FET biosensor (ZnO nanoparticle film)	3.24	-	[121]
	Nanowire field-effect transistor biosensor (NWFET)	-	100–1 × 10^4^	[131]
	Extended gate field-effect transistor (EGFET) pH Sensor (TiN sensitive film+antibody functionalization)	-	0.01–100	[123]
ECLIA	Dual-wavelength ratio-type ECL biosensor (Au-CNN+ECL-RET)	3.94 × 10^−3^	1 × 10^−5^–10	[124]
	“Signal-on” type ECL immunosensor (RuMOFNSs luminescent body)	4.8 × 10^−4^	1 × 10^−6^–10	[125]
	A three-electrode ratio-type ECL immunosensor (ABEI@GQDs nano-luminescent bodies)	3.5 × 10^−4^	1 × 10^−6^–5 × 10^−3^	[126]
	Sandwich-type ECL immunosensor (MoS_2_@Cu_2_O-Ag+Ce:ZnO@NGQDs)	2.9 × 10^−3^	0.01–100	[128]

**Table 3:** Comparison chart of other cTnI detection methods.

Techniques	Sensor	LOD (pg/mL)	Detection Range (ng/mL)	References
LFIA	Magnetic bead-assisted lateral flow immunoassay (LFIA)	100	-	[144]
	Paper/PVA hybrid lateral flow immunoassay (LFIA)+smartphone reading	0.92	-	[132]
	Quantum dot microsphere@SiO_2_-COOH nanoprobes lateral flow immunoassay (LFIA)	36	0.12–125	[133]
	Branch-shaped metal film (HD–nanometal) fluorescence lateral flow immunoassay (LFIA)	-	-	[134]
MFT	Portable plasma microfluidic biosensor (gold nanoparticle integration)	15	-	[145]
	Enzyme-linked DNA aptamer microfluidic platform (magnetic bead chemiluminescence detection)	12	-	[135]
	Electrochemical impedance spectroscopy (EIS) microfluidic chip	1000	-	[136]
	Flexible laser-induced graphene (LIG) microfluidic electrochemical biosensor	45.33	-	[137]
	Single-channel finger pump microfluidic chip (SERS+sandwich immunoassay)	10	-	[138]
IST	Double-layer high electron mobility transistor (HEMT) sensor	10	-	[140]
	Quartz crystal microbalance (QCM) immunosensor (TiO_2_photocatalytic silver staining)	18	-	[141]
	Distance-based paper-based analytical device (dPADs) non-immunoassay	25	0.025–2.5	[145]
	DNA walker—blood glucose meter (PGM) portable biosensor	1	-	[142]
	Paper-based vertical flow detection (hs-VFA) platform (deep learning enhanced)	0.2	-	[143]

## Data Availability

Not applicable.

## Abbreviations

CVDs	Cardiovascular diseases
AMI	Acute myocardial infarction
cTnI	Cardiac troponin I
anti-cTnI	Anti-cardiac troponin I antibody
cTnT	Cardiac troponin T
CRP	C-reactive protein
IFAL	Immunofluorescence analysis
ELISA	Enzyme-linked immunosorbent assay
SERS	Surface-enhanced Raman scattering
SPR	Surface plasmon resonance
LSPR	Localized surface plasmon resonance
FOST	Fiber-optic sensor technology
EIT	Electrochemical immunosensing
EADT	Electrochemical aptamer detection
FETs	Field-effect transistors
POC	Point-of-care
POCT	Point-of-care testing
ECLIA	Electrochemiluminescence immunoassay
LOD	Limit of detection
PEC	Photoelectrochemical sensing
afGQDs	Amine-functionalized graphene quantum dots
ZIF-8	Zeolitic imidazolate framework-8
Ab2	Secondary antibody
COU	Coumarin
BSA	Bovine serum albumin
CEA	Carcinoembryonic antigen
AFP	Alpha fetoprotein
MPA	Mercaptopropionic acid
OPD	o-phenylenediamine
oxOPD	Oxidized to 2,3-diaminophenazine
AP	4-aminophenol
EA	Ethylenediamine
PCDs	Polymer carbon dots
ALP	Alkaline phosphatase
RSD	Relative standard deviation
GPRu-Au	Au nanoparticle-loaded graphene oxide/polyethylenimine
aptHCR	Aptamer-based DNA nanostructure produced by hybridization chain reaction
